# Is Austerity Responsible for the Stalled Mortality Trends Across Many High-Income Countries? A Systematic Review

**DOI:** 10.1177/27551938241255041

**Published:** 2024-05-20

**Authors:** Philip Broadbent, David Walsh, Srinivasa Vittal Katikireddi, Christine Gallagher, Ruth Dundas, Gerry McCartney

**Affiliations:** 1 47970 University of Glasgow MRC/CSO Social and Public Health Sciences Unit, Glasgow, UK; 2 347493 University of Glasgow School of Health and Wellbeing, Glasgow, UK; 3 578987 Public Health Scotland Glasgow Office, Glasgow, UK; 4 150729 University of Glasgow College of Social Sciences, Glasgow, UK

**Keywords:** austerity, mortality, life expectancy, lifespan variation, mortality rate

## Abstract

This article systematically reviews evidence evaluating whether macroeconomic austerity policies impact mortality, reviewing high-income country data compiled through systematic searches of nine databases and gray literature using pre-specified methods (PROSPERO registration: CRD42020226609). Eligible studies were quantitatively assessed to determine austerity's impact on mortality. Two reviewers independently assessed eligibility and risk of bias using ROBINS-I. Synthesis without meta-analysis was conducted due to heterogeneity. Certainty of evidence was assessed using the GRADE framework. Of 5,720 studies screened, seven were included, with harmful effects of austerity policies demonstrated in six, and no effect in one. Consistent harmful impacts of austerity were demonstrated for all-cause mortality, life expectancy, and cause-specific mortality across studies and different austerity measures. Excess mortality was higher in countries with greater exposure to austerity. Certainty of evidence was low. Risk of bias was moderate to critical. A typical austerity dose was associated with 74,090 [−40,632, 188,792] and 115,385 [26,324, 204,446] additional deaths per year. Austerity policies are consistently associated with adverse mortality outcomes, but the magnitude of this effect remains uncertain and may depend on how austerity is implemented (e.g., balance between public spending reductions or tax rises, and distributional consequences). Policymakers should be aware of potential harmful health effects of austerity policies.

In the decade preceding the COVID-19 pandemic, trends in mortality (and related measures such as life expectancy) in many high-income countries slowed and in some cases reversed, departing from previous patterns of continual improvement.^1–4^ The changed trends have been unequally experienced such that mortality rates are actually increasing for those living in the most deprived areas of the United States, England, Wales and Scotland, leading to widening health inequalities.^5–7^

In many countries the changed trends in mortality and life expectancy occurred shortly after austerity policies were implemented in the wake of the 2007–2008 financial crisis and subsequent economic recession.^8^ While governments may have limited ability to prevent recessions, austerity itself is a purposive policy aim. Whether austerity could have played a causal role in the described changes in life expectancy is subject to considerable debate.^9–11^

Most definitions of austerity share a commonality of “fiscal consolidation:” intentional policy aimed at reducing government debts and debt accumulation with the goal of “creating space” for the private sector to grow.^12–16^ However, fiscal consolidation and austerity are not synonymous, as (arguably) austerity should be reserved for the implementation of fiscal consolidation during economic downturns.^17^ Measures of austerity are subtly different in what is captured, and the extent to which this accounts for economic growth and the changes in taxes and spending that arise independently of policy changes as a consequence.^18^ Austerity policies involve some combination of reduced public expenditure or increased taxation, but this varies between countries, both in nature and extent. In the United Kingdom, for example, austerity policies resulted in reductions in the real-term value of many social security benefits, as well as increased constraints on their conditionality and eligibility.^19,20^ Furthermore, there were large cuts to local government spending^21^ and a slower rate of increase in health care spending.^22^ This limits the ability of local governments to fund services such as housing, education, leisure, and social care services. A growing body of literature suggests that such changes (in combination with other mechanisms) could have detrimental impacts on population health and wellbeing, including negatively impacting on mortality and life expectancy.^23–25^ Furthermore, these effects may not be experienced equally.^5^ As well as inequalities in life expectancy trends by socioeconomic position, austerity measures in the United Kingdom and in other countries have coincided with widening income inequalities in both absolute and relative terms.^26,27^

Existing research indicates that economic influences play an important role in determining trends in population mortality outcomes acting, for example, through pathways such as changes in health behaviors (e.g., smoking and excessive alcohol use) or the availability and affordability of material resources for living, such as housing.^28,29^ However, there is a lack of consensus within the literature as to whether economic recession specifically has overall beneficial or detrimental impacts on population health (as measured, for example, by mortality rates).^30^ In fact, there is some evidence to suggest that economic recession is actually associated with improvements in all-cause mortality and most cause-specific mortality rates except suicide.^27^ Furthermore, improvements in life expectancy trends have been seen during previous economic recessions, suggesting that there must be another mechanism driving the present stalling of life expectancy and mortality improvements.^11^ Distinct consideration of the impact of austerity is therefore imperative.

Although much is known about the impact of political decision-making and the wider political economy on population health outcomes,^28^ there has not yet been a systematic review investigating the association between macroeconomic austerity policies and population mortality outcomes. We hypothesize that post-2008 fiscal austerity policies have negatively impacted mortality rates in high-income countries. This article will review the evidence of the relationship between austerity policies and mortality trends in high-income countries to test this hypothesis and clarify the effects of austerity measures on population health.

## Methods

### Overview

The study adheres to the Preferred Reporting Items for Systematic Review and Meta-Analysis (PRISMA^31^;) and Synthesis Without Meta-analysis (SWiM^32^;) reporting guidance. The protocol for this study is registered with PROSPERO (registration number CRD42020226609). Any deviation from the protocol is reported in the appendix.

### Literature Search

We conducted a comprehensive literature search of peer-reviewed and gray literature, including economics working papers and academic theses, to identify studies investigating the effect of austerity policies on mortality outcomes. The search was performed using the following databases: OVID Medline, EMBASE, Proquest Public Health, Proquest Sociological Abstracts, Proquest Applied Social Sciences Index and Abstracts (ASSIA), Scopus, Web of Science, The Cochrane Central Register of Controlled Trials (CENTRAL), and PROSPERO. The search strategy was developed in consultation with an information specialist and is included in the appendix. We initially conducted the search in January 2021, with searches updated in November 2022. No date limits were applied to the search.

### Inclusion Criteria

Table 1 illustrates the inclusion criteria used. The population of interest was individuals living in high-income countries according to the World Bank's definition of countries who had a gross national income per capita greater than or equal to US $12,696 in 2020,^33^ and the exposure of interest was the implementation of austerity policies. We included randomized and non-randomized quantitative studies, with the latter category including any non-randomized study design provided comparison was made between an exposed and unexposed (or less exposed) group. References were de-duplicated and imported into “Covidence” software for screening. The initial search was conducted in January 2021. To maintain the currency of the systematic review, an updated search was conducted in November 2022, incorporating any new studies meeting the inclusion criteria. This step ensured that the review included the most recent and relevant evidence available.

**Table 1.** Population, Exposure, Comparator, and Control (PECO) Criteria Used in This Systematic Review.

Population of interest	Any national-level population, of any age group, at any time period, of high-income countries (defined according to the World Bank as countries whose “economies are those with a GNI per capita of US$12,696 or more in 2020;” 33).
Exposure	Any quantitative summary indicator of overall, intentional austerity measures enacted by governments. This definition is intended to reflect measures that reflect the comprehensive fiscal stance, rather than alterations to individual taxes or specific departmental expenditures. Studies that only compared mortality rates before and after the implementation of austerity measures, without quantitatively linking the extent of austerity to mortality outcomes, were omitted. Our definition of austerity was not confined to the economic downturn periods.
Comparator/control	To provide variation in the exposure measure(s), only studies including some form of comparison were included: In the same population before and after implementation of austerity Between countries employing differing degrees of austerity A combination of the above
Outcome	Mortality (or mortality-derived measure), either cause-specific or all-cause mortality acceptable. Mortality measures of specific sub-populations (e.g., infant mortality rate) were accepted.
Study design	Randomized and non-randomized quantitative studies (including longitudinal cross-sectional and natural experiment studies).
Time period	All time periods were considered.

### Study Selection

All titles, abstracts and full-text articles were independently screened by two reviewers, with conflicts resolved by consensus or discussion with a third reviewer. Non-English studies were excluded at the full-text stage. A list of all articles excluded at the full-text stage of screening is included in the appendix. Reference lists of relevant systematic reviews and included studies were screened for additional studies. Where eligible studies had more than one reason for exclusion, a set of decision rules was considered, which included alignment with our PECO and risk of bias criteria.

### Data Extraction and Risk of Bias Assessment

Using a standardized template, data on key study characteristics, exposure details and reported outcome data were extracted from each eligible study identified in the systematic search. We accounted for instances where the reporting of some studies did not encompass all results, carefully examining the underlying data to ensure a comprehensive review. The impacts of austerity were summarized both quantitatively and narratively. The Cochrane Risk of Bias in Non-Randomised Studies – of Interventions (ROBINS-I) tool was used to assess potential bias for each study by one reviewer and checked by a second.^34^ ROBINS-I has a theoretical framework grounded in counterfactual reasoning, which is increasingly fundamental in modern epidemiological research.^35,36^ The tool, which is applicable to various study designs, evaluates the risk of bias for non-randomized studies by comparing them against a hypothetical randomized trial, a “target trial.” This approach is considered a significant improvement over previous methods for assessing evidence from non-randomized studies, as it provides a more rigorous and structured framework for evaluating the risk of bias in causal inferences.^37–39^ In line with the guidance, the overall risk of bias for a study was determined by the domain with the greatest risk of bias. Further details on the data extraction and risk of bias assessment are available in the appendix.

### Data Synthesis

Synthesis Without Meta-Analysis. Due to the heterogeneity of the data, a meta-analysis was not feasible. We presented an effect direction plot for all included studies, in line with Cochrane guidance, and statistical significance was not considered during this classification.^40^

Due to the variability in the measurement of exposure (i.e., different austerity measures), the timing of the exposure (effects measured at different time lags), and the population structure (e.g., gender-specific outcomes), we did not calculate pooled effect estimates for groups of outcome measures.

We used the modeling coefficients provided in each of the original studies to construct forest plots. These plots were organized based on the specific mortality outcome measure and were further stratified according to the austerity measure applied in each study. This approach allowed us to visually represent and compare the effects across different studies, even with the variations in their austerity measures.

The interpretation of these coefficients remains outcome-specific. For instance, for age-standardized mortality rates (ASMR), cause-specific mortality rates, and lifespan variation, a positive coefficient indicates that an increase in austerity measures is correlated with negative health impacts. This implies an increase in mortality rates or a variation in lifespan. Conversely, for life expectancy outcomes, a negative coefficient suggests detrimental effects, denoting a decline in life expectancy with increased austerity. Coefficients are presented to two significant figures.

#### Certainty Assessment

The overall certainty of evidence contained within the synthesis was assessed using the Grading of Recommendations Assessment, Development and Evaluation (GRADE) framework,^41^ which considers five domains: risk of bias, imprecision, inconsistency, indirectness, and publication bias. Key outcomes focused on the impact of austerity on mortality outcomes. Each domain was assessed independently by the author and a second reviewer according to these criteria, with consensus regarding certainty of evidence agreed by discussion. Absolute effect estimates for the United Kingdom were calculated by applying effect estimates from the source studies to the Office of National Statistics mid-2021 population estimates in order to provide a more tangible understanding of the impact of austerity on mortality outcomes, enabling clearer interpretations.^42^ Different austerity measures operate on varied scales, making comparisons challenging. Standard deviations were not reported in several primary studies meaning standardization across scales was not possible. Therefore, for clarity and consistency, we employed the cyclically adjusted primary balance (CAPB)—a standard measure in the literature linked directly to our key mortality outcomes. We specifically measure the impact of an austerity change, as measured by CAPB, equivalent to the United Kingdom's “dose” in 2010, marking the onset of austerity policies in the United Kingdom. A summary of the certainty of evidence and absolute effect measures is presented in a condensed summary of findings table, with further details provided in Appendix VII.^43^

## Results

**Figure 1.**
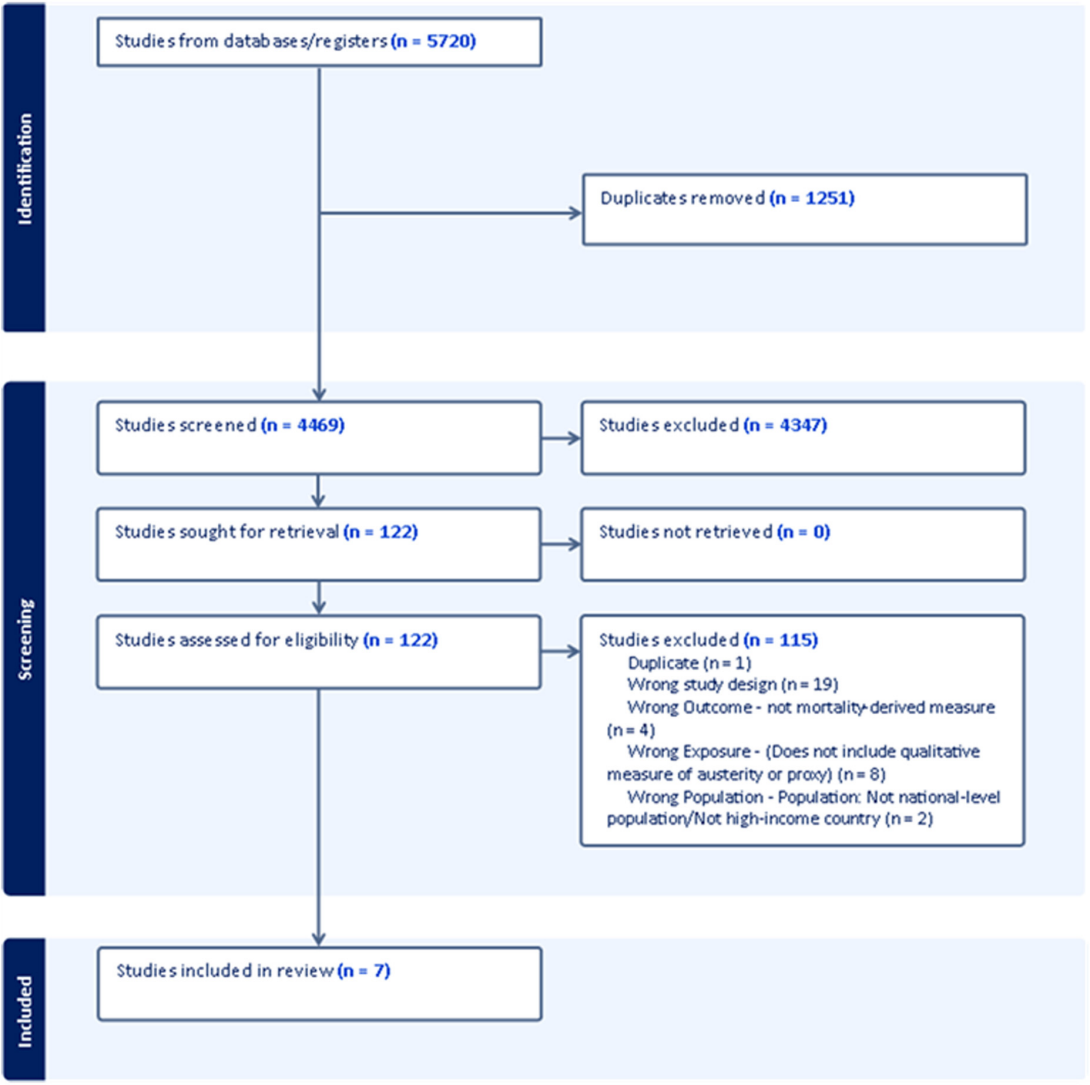
PRISMA flowchart illustrating process of identification, screening, and selection.

Of 5,720 studies screened, seven were eligible for inclusion. All included studies used whole-population administrative datasets (see Figure 1 for details regarding the screening process and Table 2 for details of all included studies and Appendix for excluded studies).

**Table 2.** Details of Included Studies.

Authors	Publication Year	Study Years	Number of countries studied	Number of data coefficients	Study Design	Measurement of austerity (exposure)	Measurement of mortality (outcome)	Overall risk of bias assessment
Predkiewicz et al.	2022	1995– 2019	21	2	Panel analysis	GGE	Life expectancy	Moderate
McCartney et al.	2022	2000– 2019	37	71	Panel analysis	CAPB, AAFI and RGE	Life expectancy Age-standardized mortality rate Lifespan variation	Moderate
Toffoluti et al. ^44^	2019	1991– 2013	28	5	Panel analysis	AAFI	All-cause mortality rate Cause-specific mortality rates	Moderate
Rajmil et al. ^45^	2019	2011– 2015	15	4	Panel analysis	CAPB	Excess mortality	Moderate
Rajmil et al. ^46^	2018	2005– 2015	16	2	Panel analysis	CAPB	Infant mortality rate	Serious
Green ^47^	2017	2011– 2015	1	1	Cross-sectional analysis	TGE	Life expectancy	Critical
Antonakakis et al. ^48^	2015	1968– 2012	5	6	Panel analysis	TGE	Suicide mortality rate	Serious

CAPB: Cyclically adjusted primary balance.

AAFI: Alesina-Ardagna Fiscal Index.

GGE: General government expenditure as a percentage of GDP.

TGE: Total government expenditure as a percentage of GDP.

RGE: Real government expenditure.

Overall risk of bias assessment scale includes low, moderate, serious, and critical.

### Exposure Measurements

Five measures were employed across the included studies to assess the impact of austerity on health outcomes: CAPB, total government expenditure (TGE) as a percentage of gross domestic product (GDP), real government expenditure (RGE) as a percentage of GDP, general government balance (GGB), and the Alesina-Ardagna Fiscal Index (AAFI).

TGE and RGE focus on the expenditure side of government finances, with RGE adjusting for inflation, while TGE does not. On the other hand, GGB considers both revenue and expenditure, providing a more comprehensive view of the government's fiscal position.

CAPB and AAFI adjust for economic fluctuations and automatic stabilizers. Automatic stabilizers refer to mechanisms that automatically adjust government spending and revenues in response to fluctuations in economic activity without the need for additional legislation or intervention. For instance, unemployment benefits may increase as unemployment rises. Adjusting for automatic stabilizers allows for a more precise measurement of discretionary government fiscal policy, separating it from the cyclical changes in expenditure and revenue that occur due only to changes in economic activity.

CAPB isolates changes resulting from government decision-making, while AAFI further adjusts for changes in asset prices, providing a more nuanced understanding of the influences on government revenues and expenditures.

Among the studies we reviewed, TGE was the sole measure of austerity in two studies, CAPB in two studies, AAFI in one study, and GGB in another. One study used AAFI, RGE, and CAPB to triangulate the effect of austerity.

### Outcome Measurements

In the included studies, five different outcome measures were used. Period life expectancy was used in three studies, providing 32 data points. Excess mortality estimates (calculated by comparing the observed number of deaths with the expected number of deaths based on past trends) were used in two studies providing two data points. Cause-specific mortality rates, which provide information about the relative burden of different causes of death in a population, were used in two studies providing 13 data points. All-cause mortality, a measure of the total number of deaths due to any cause in a population, was used in three studies providing 29 data points. Lifespan variation, which measures the variation in expected lifespan across a population, and is considered a proxy measure for socioeconomic inequalities in mortality outcomes,^49^ was used in one study providing 27 data points.

### Risk of Bias Assessment

Out of the seven included studies, two were at critical risk of bias, one at serious risk of bias, and four at moderate risk of bias, with no studies deemed low risk of bias (i.e., equivalent to a well-conducted randomized controlled trial). Confounding was identified as the risk of bias domain in which most studies performed poorly (for domains of bias, see the online appendix 2).

The studies by Toffoluti and colleagues,^44^ Rajmil and colleagues,^45^ McCartney and colleagues,^50^ and Predkiewicz and colleagues^51^ used a panel analysis design to eliminate the impact of time invariant confounders (such as welfare state type). These authors also used a measure (or measures) of austerity which controlled for automatic stabilizers (including unemployment, a key marker of economic recession). However, these measures may not eliminate the confounding from other time varying confounders.

The studies in our review captured data from periods that experienced economic recessions, with some studies covering multiple recessionary periods, such as Toffolutti (1991–2013) and Antonokakis (1968–2012). This is an important consideration, as “historical aggregate economic activity” was identified as a key confounder in our protocol. Evidence regarding the impact of economic recession on mortality outcomes is inconclusive, and it is reasonable to assume that major economic events, including recessions, could have influenced mortality outcomes during these periods, independent of austerity policies.

The included measures of austerity account for changes in GDP, which is one method of adjusting for the potential confounding effects of economic recession. This adjustment is likely to account for some of the confounding, but there may still be residual confounding that remains unaddressed. Moreover, while some studies in our review cover multiple recessionary periods, which may help to better capture the recessionary effects, the adequacy of the GDP adjustment may vary across studies and recessionary contexts. One study also undertook sensitivity analyses which restricted the dataset to periods of economic downturn only, comparing countries which did and did not implement austerity in such periods, as a further attempt to isolate the discrete impact of austerity policies,^50^ although we have not included these data in our synthesis.

It is important to note that statistical adjustment alone is often insufficient to achieve a low risk of bias, as evidenced by the fact that most randomized trials are also not assessed as low risk. We therefore deemed the included studies to be at moderate risk of bias. This assessment acknowledges the efforts to adjust for GDP changes while also recognizing the potential for residual confounding and the limitations posed by the varying contexts of different recessionary periods.

The study by Rajmil and colleagues^46^ was deemed to be at overall serious risk of bias due to confounding and missing data. Although the studies by Antonakakis and colleagues^48^ and Green^47^ used a measure of austerity which was calculated as a percentage of GDP (thereby partially accounting for recession and economic growth effects), this measure did not account for automatic stabilizers. The Green study was also subject to risk of time invariant confounding (e.g., welfare state type). Both studies were deemed to be at overall critical risk of bias. Individual risk of bias assessments for each study are provided in the online appendix 2.

### Effect Direction

**Figure 2.**
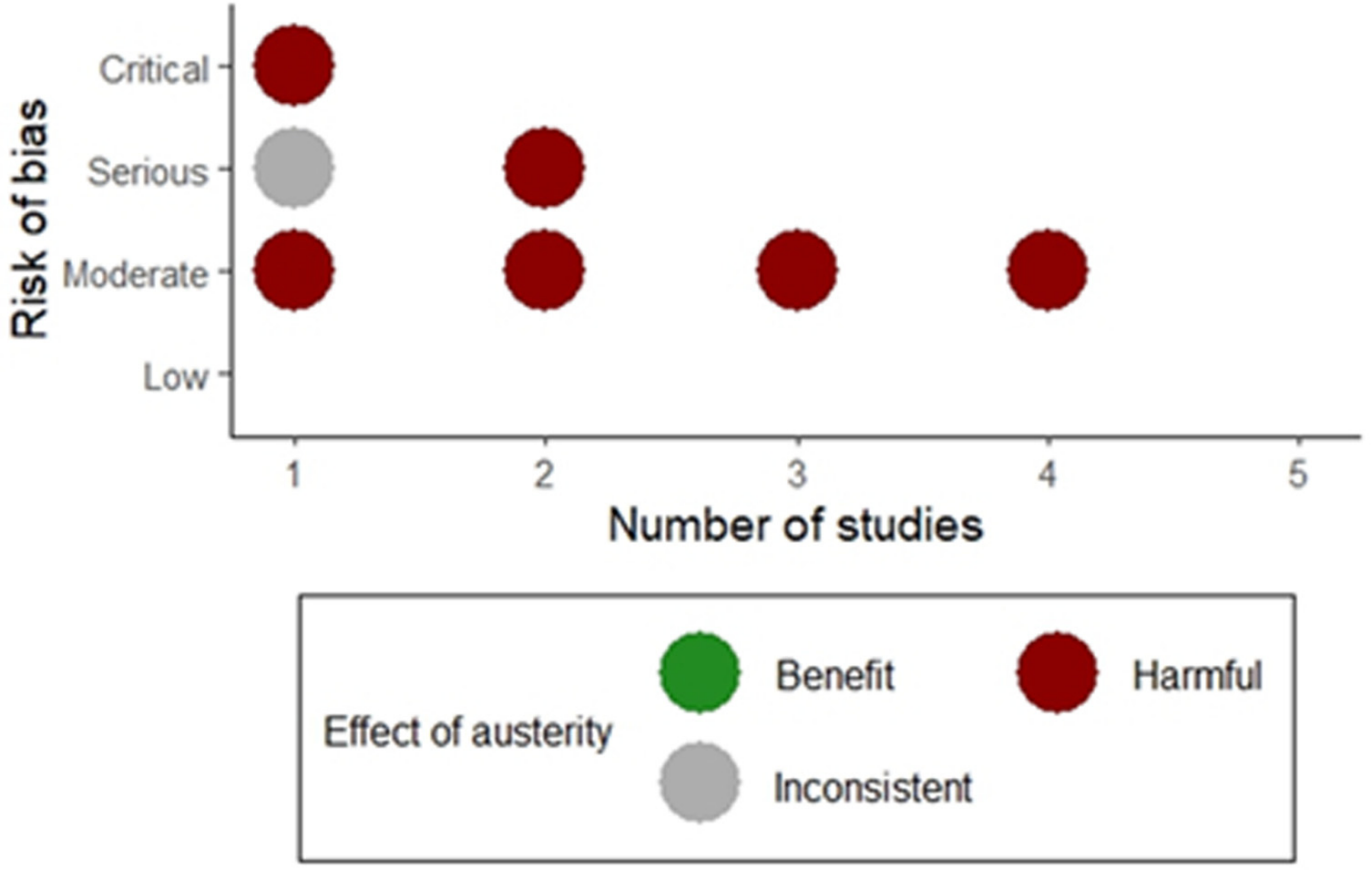
Effect direction plot considering the impact of austerity policies.

The effect direction for all the included studies, stratified by risk of bias, is summarized in Figure 2, showing that austerity policies had harmful impacts on mortality outcomes in six out of seven of the studies, including the four studies at lowest risk of bias.

### Effects on Mortality Outcome Measures

The results demonstrate the impact of a one-unit change in austerity measures on the change in the standard deviation of the mortality measure in question. The effects vary depending on the specific measure of austerity and time lag.

#### Age-Standardized Mortality Rate

**Figure 3.**
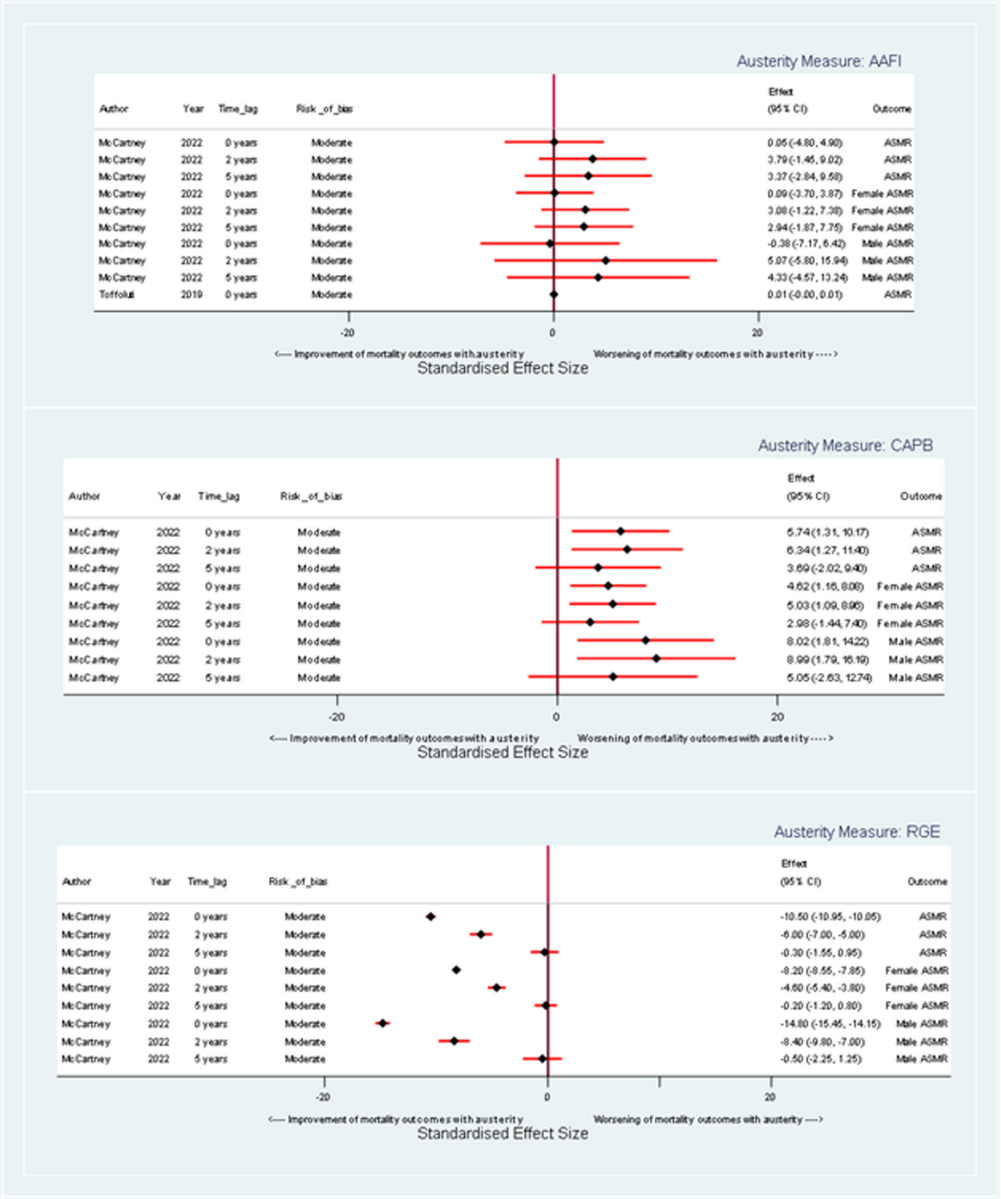
Forest plot illustrating the effect of a one-unit change in austerity on age-standardized mortality rate coefficients, stratified by austerity measure.

Using the AAFI measure, the results indicate an association between a one-unit increase in austerity and a harmful effect on ASMR for both males and females, with the effect becoming more pronounced at longer time lags (see Figure 3). At a 0-year time lag, the coefficient for overall ASMR is 0.05 (95% confidence interval [CI]: −4.80,4.90) suggesting an equivocal impact of austerity. For female ASMR, the coefficient is 0.09 (95% CI: −3.70, 3.87), and for male ASMR, it is −0.38 (95% CI: −7.67, 6.43). At a 5-year time lag, the coefficients for overall ASMR increase to 3.37 (95% CI: −2.84, 9.58) with female and male ASMR coefficients being 2.94 (95% CI: −1.87, 7.75) and 4.33 (95% CI: −4.57, 13.24), respectively suggesting harmful but imprecise effects of austerity.

When assessed by the CAPB measure, a unit increase in austerity is again linked with increased ASMR for both genders, but this effect lessens with increasing time lag between exposure and outcome (see Figure 3). At a 0-year time lag, the coefficient for overall ASMR is 5.74 (95% CI: 1.31, 10.37), and the coefficients for males and females are 8.02 (95% CI: 1.81, 14.22) and 4.62 (95% CI: 1.16, 8.08) respectively. By a 5-year time lag, the coefficient for overall ASMR lessens to 3.69 (95% CI: −0.57, 2.91), with coefficients for males and females at 5.05 (95% CI: −2.63, 12.74) and 2.98 (95% CI: −1.44, 7.40), respectively. Overall, there is a consistent harmful, but imprecise, effect of austerity.

Using the RGE measure, there is a harmful impact on ASMR for males and females and the overall population that reduces over time (see Figure 3). For a unit increase in austerity as measured by RGE at a 0-year time lag, the coefficient for change in overall ASMR is 10.50 (95% CI: 10.05, 10.95), indicating a harmful impact of austerity. For female ASMR, the coefficient is 8.20 (95% CI:7.85, 8.55), and for male ASMR, it is 14.80 (95% CI:14.15, 15.45). By a 5-year time lag, the coefficient for change in overall ASMR per unit increase in austerity is 0.30 (95% CI: −0.95, 1.55), with female and male ASMR coefficients at 0.20 (95% CI: −0.80, 1.20) and 0.50 (95% CI: −1.25, 2.25), respectively. Overall, there is a harmful effect of austerity which tends to zero after 5 years.

Overall, regardless of measure of austerity, the results indicate that a unit increase in austerity is associated with an increase in ASMR, which is harmful for both sexes and the overall population. The magnitude and trajectory of these effects vary depending on the specific austerity measure and the time lag being considered, but the greatest impacts appear among males at a 0-year time lag when austerity is measured using RGE.

#### Life Expectancy

**Figure 4.**
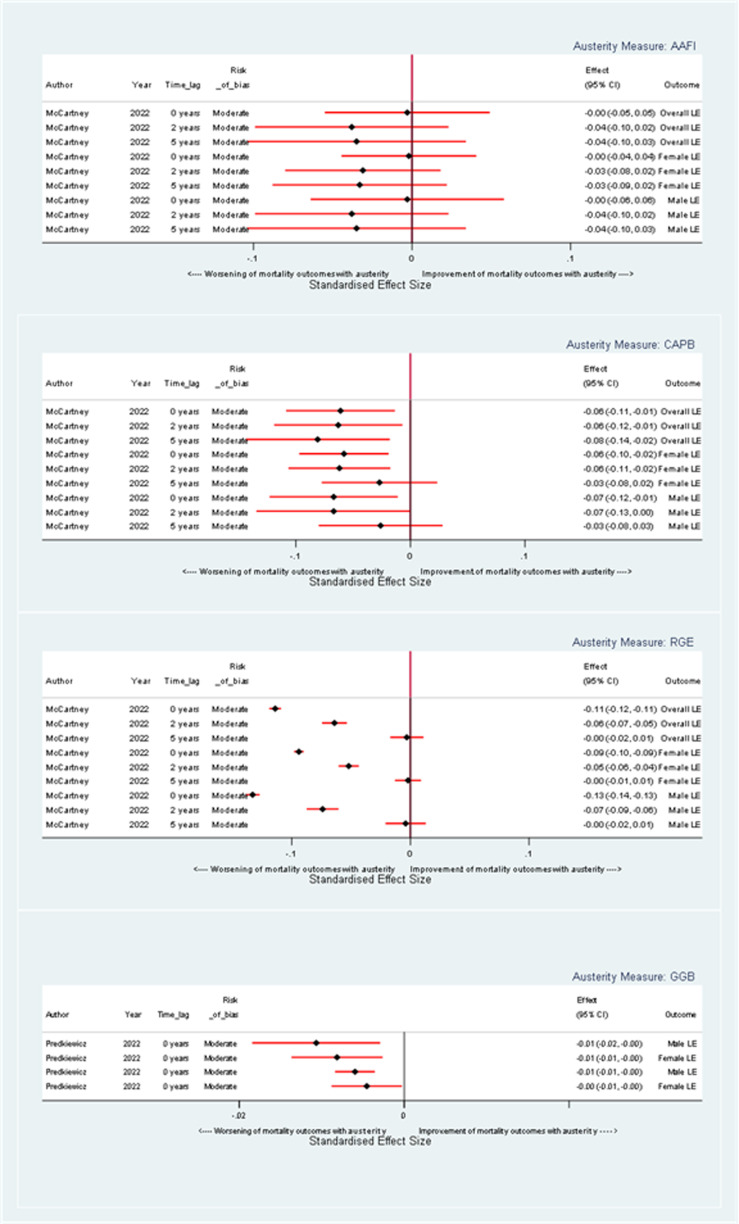
Forest plot illustrating the effect of a one-unit change in austerity on life expectancy coefficients, stratified by austerity measure.

Corresponding with findings on age-standardized mortality rate, for each unit increase in austerity as measured by AAFI, there is evidence of a harmful effect on life expectancy, which increases over time (see Figure 4). At a 0-year time lag, the coefficient for overall life expectancy is equivocal at 0.00 years (95% CI: −0.055, 0.049). At a 5-year time lag, the coefficient for overall life expectancy is −0.04 years (95% CI: −0.10, 0.03). For female life expectancy, the coefficients at 0 and 5-year time lags are 0.00 years (95% CI: −0.05, 0.04), −0.031 and −0.03 years (95% CI: −0.09, 0.02) respectively. For male life expectancy, the coefficients are 0.00 years (95% CI: −0.06, 0.06) and −0.03 years (95% CI: −0.10, 0.03) at 0 and 5-year time lags, respectively.

Similarly, when austerity is measured by CAPB, there is a harmful effect on life expectancy (see Figure 4). At a 0-year time lag, the overall life expectancy showed a decrease with a coefficient of −0.06 years (95% CI: −0.11, 0.01) with coefficients for female and male life expectancy of −0.06 years (95% CI: −0.10, −0.02) and −0.07 years (95% CI: −0.12, −0.01) respectively. These effects appear more pronounced in the short term and gradually diminish with longer lag times.

When measured by RGE, a unit increase in austerity has a harmful effect on overall life expectancy, with a coefficient of −0.11 years (95% CI: −0.12, −0.11) at a 0-year time lag (see Figure 4). Corresponding coefficients for males and females are −0.09 years (95% CI: −0.11, −0.07) and −0.13 years (95% CI: −0.15, −0.11). This harmful impact reduces over time so that the impact of austerity is close to equivocal for both genders and, overall, at a 5-year time lag.

When measured by GGB, austerity has a harmful impact on both male and life expectancy at 0- and 1-year time lags (see Figure 4). For example, the coefficient for male life expectancy at both 0- and 1-year time lags per unit increase in austerity as measured by GGB is 0.01 years.

In the article by Green and colleagues (47; data not included in Figure 4), the correlation coefficient of 0.49 between government expenditure and life expectancy suggests an association where decreased spending correlates with lower life expectancy. However, the specific quantitative decrease in life expectancy per unit reduction in government spending is not reported.

In summary, regardless of the measure used, a unit increase in austerity has a harmful effect on life expectancy. The magnitude and pattern of these effects vary, depending on the specific austerity measure, gender, and time lag under consideration.

#### Excess Mortality

Rajmil and colleagues^45^ used a categorical measure to examine the impact of austerity on all cause excess mortality. They found that, in comparison with countries in their “low” tercile of austerity, countries in “medium” austerity tercile experienced excess mortality of 40.2 per 100,000 per year (95% CI: 9.32, 71.08) and countries in the “high” austerity tercile had an overall excess mortality of 31.2 per 100,000 per year (95% CI: 0.34, 62.1). The article also conducted two generalized estimating equation (GEE) models to evaluate the impact of austerity on standardized mortality rates (SMRs). Model 1 measured the direct effect of transitioning from low to higher austerity levels (intermediate and high) on SMRs over the period 2011–2015. Model 2 added a temporal dimension, treating specific years within this timeframe as an independent variable, to examine annual changes in the austerity-SMR relationship. In Model 1, moving from low to intermediate austerity increased SMRs by 50.1 per 100,000 per year (95% CI: −116.7, 16.5), and to high austerity by 22.7 per 100,000 per year (95% CI: −89.3, 44.0). In model 2 moving from low to intermediate or high austerity was related to an increase of 70.0 per 100,000 per year (95% CI: −139.4, −0.6) and 35.2 per 100,000 per year (95% CI: −104.6, 34.3) respectively.

#### Infant Mortality

A categorical measure was also used comparing “high,” “medium,” and “low” terciles of austerity in the study by Rajmil and colleagues.^46^ This study found that infant mortality rates showed a continuous decreasing trend across three austerity terciles over the study, with rates in low austerity countries reducing by 0.48 percent per year and by 0.40 percent in high austerity countries. However, using a GEE model to account for differing secular trends, it was found that austerity, as measured by CAPB, had no significant effect on infant mortality rates for the periods 2008–2010 and 2012–2015, with coefficients of −0.002 and 0.10 respectively.

#### Cause-Specific Mortality Rates

**Figure 5.**
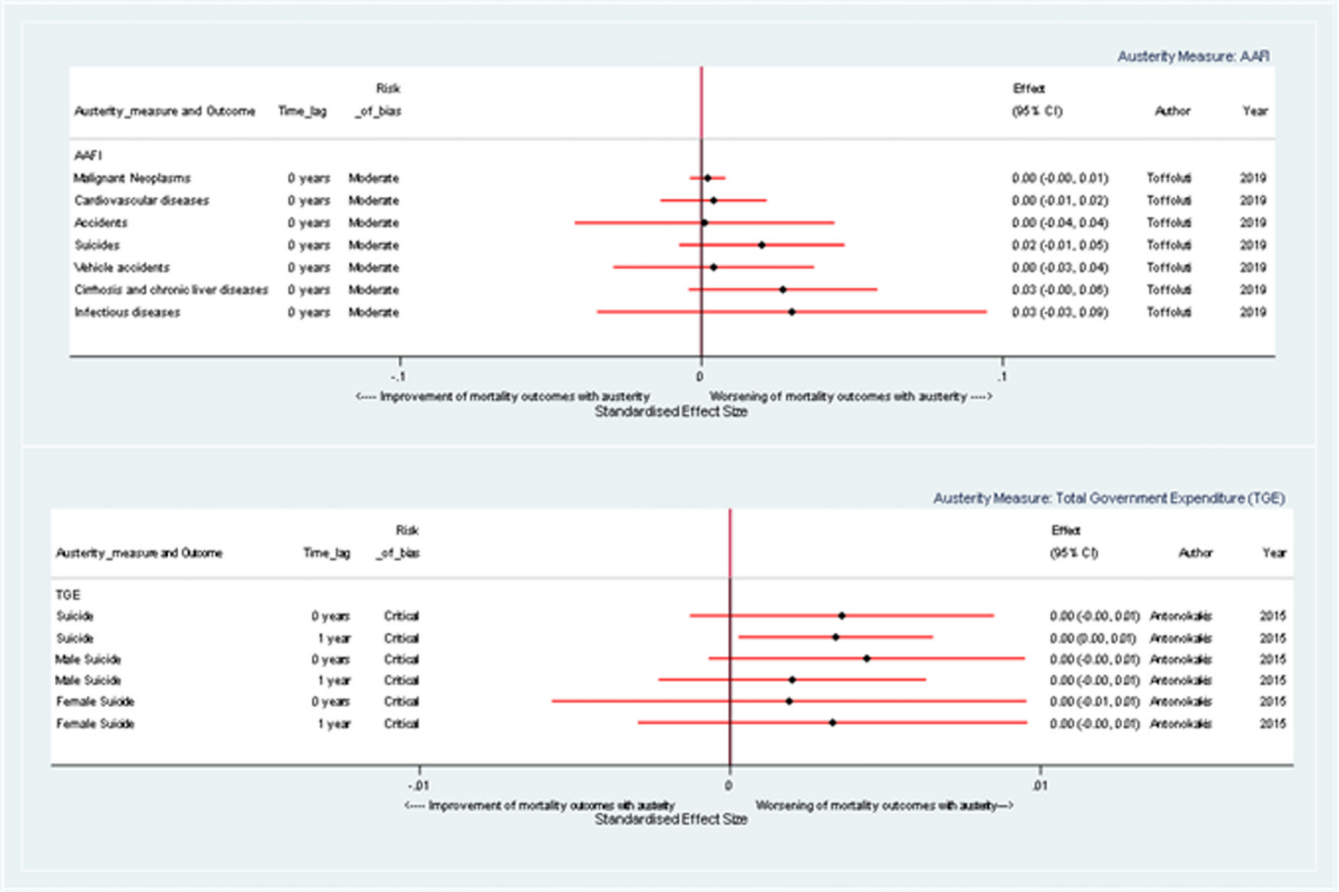
Forest plot illustrating the effect of a one-unit change in austerity on cause-specific mortality rate coefficients, stratified by austerity measure.

Figure 5 illustrates the change in the coefficients for cause-specific mortality, resulting from a unit change in austerity measures, and demonstrating that austerity typically has detrimental impacts on mortality across a wide array of causes.

For instance, when austerity is gauged by AAFI, positive coefficients are demonstrated for mortality due to malignant neoplasms (0.20% [95% CI: −0.39, 0.79]), cirrhosis and chronic liver diseases (2.70% [95% CI: −0.44, 5.84]), and suicides (2.00% [95% CI: −0.74, 4.74]), indicating a harmful effect. Similarly, when measured by TGE, there appears to be an association between austerity and male, female, and overall suicide mortality coefficients, suggesting a harmful impact. For example, the coefficients for change in overall suicide mortality are 0.36 percent (95% CI: −0.13, 0.85) and 0.34 percent (95% CI: 0.03, 0.65) at 0- and 1-year time lags respectively.

The effect of austerity on cause-specific mortality depends on the measure used, the time lag observed, and the specific cause of death. Overall, however, the results suggest that an increase in austerity is generally associated with harmful effects, but these estimates are very imprecise.

#### Lifespan Variation

**Figure 6.**
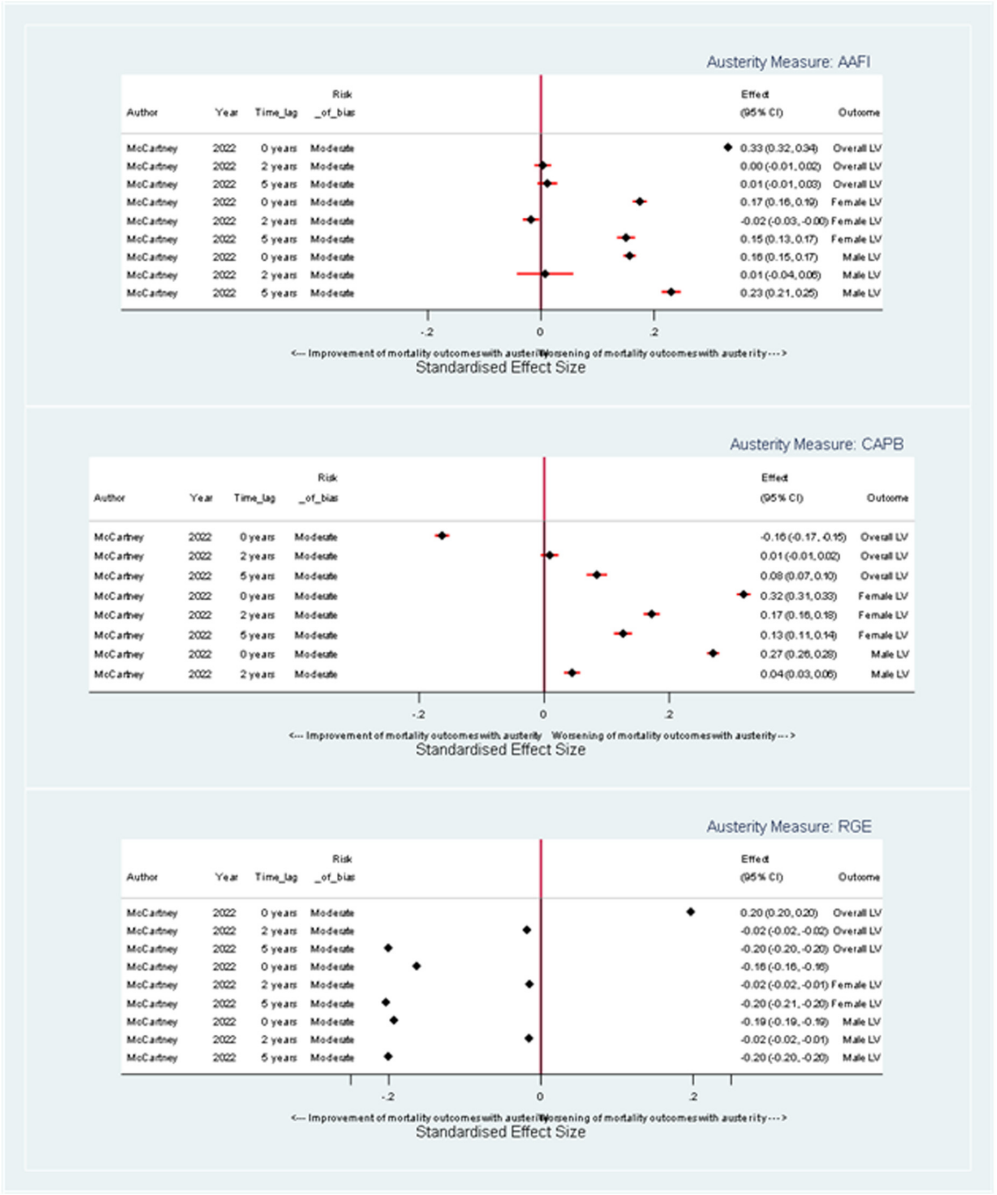
Forest plot illustrating the effect of a one-unit change in austerity on lifespan variation coefficients, stratified by austerity measure.

Figure 6 illustrates the coefficients for lifespan variation resulting from a unit change in austerity measures. The observed effects vary according to the specific measure of austerity and the time lag.

When austerity is measured by AAFI measure and with no time lag, there is a coefficient of −0.002 years (95% CI: −0.008, 0.004) for overall population lifespan variation while female and male lifespan variations show coefficients of −0.004 years (95% CI: −0.016, 0.008) and 0 years (95% CI: −0.011, 0.011), respectively, indicating equivocal or possibly beneficial impacts on lifespan variation. At a 5-year time lag, the corresponding coefficients are 0.005 years (95% CI: −0.012, 0.022), 0.006 years (95% CI: −0.010, 0.022) and 0.002 years (95% CI: −0.015, 0.019), respectively (see Figure 6).

When measured by CAPB with no time lag, there is a small harmful impact on lifespan variation: 0.013 years for overall (95% CI: 0.002, 0.024), 0.018 years for females (95% CI: 0.007, 0.029), and 0.006 years for males (95% CI: −0.004, 0.016). At a 5-year lag, the impact of a unit increase in austerity shows a beneficial trend for overall and male lifespan variations (−0.003 years, 95% CI: −0.019, −0.013; −0.010 years, 95% CI: −0.025, 0.005, respectively). Notably, female estimates consistently show worse outcomes than male estimates across different time lags.

When measured by RGE, at a 0-year time lag, the coefficients for the impact of a unit change in austerity on overall, female, and male lifespan variations are 0.024 years (95% CI: 0.023, 0.025), 0.024 years (95% CI: 0.023,0.025), and 0.018 years (95% CI: 0.017,0.019), respectively, indicating a harmful impact of austerity (see Figure 6). However, this impact appears to reduce over time with the coefficients for the impact of a unit change in austerity on overall, male, and female lifespan variation close to zero at a 5-year time lag.

Overall, a unit increase in austerity, measured by AAFI, RGE, and CAPB, influences lifespan variation differently over time and by austerity measure. There does not appear to be a consistent impact of austerity on lifespan variation across the different measures of austerity.

### GRADE Assessment of Certainty of Direction of Effect of Austerity on Mortality Outcomes

In our GRADE assessment, we found that austerity has harmful effects on both ASMRs and life expectancy; however, the overall certainty of the evidence is low (see Table 3). We observed that the harmful effect of austerity on ASMRs and life expectancy was apparent in most measures across populations and time lags, but not consistently in all measures or populations. The certainty of evidence was downgraded due to risk of bias and inconsistency. When applied to the mid-2021 U.K. population, and the 2021 U.K. life expectancy and ASMR estimates, we found that the impact of a one-unit increase in austerity varied according to the time lag considered.

**Table 3.** Table of GRADE Certainty of Evidence and Estimation of the Absolute Effect of an Increase in Austerity on Mortality Outcomes for the United Kingdom Using mid-2021 Population Estimates.

Certainty assessment							Summary of findings
*Number of studies* Countries	Risk of bias	Inconsistency	Indirectness	Imprecision	Publication bias	Overall certainty of evidence	Absolute effect estimates on mortality outcomes of a −3.2 unit in CAPB^c^ when applied to the ONS UK Population estimate (mid-year 2021) of 67,026,292 [95% CI]
Age-standardized mortality rate (0-year lag)							
*1* 37	serious^a^	serious^b^	not serious	not serious	none	⨁⨁◯◯ Low	115,385 [26,324, 204,446] additional deaths per year
Age-standardized mortality rate (5 years lag)							
*1* 37	serious^a^	serious^b^	not serious	not serious	none	⨁⨁◯◯ Low	74,090 [−40,632, 188,792] additional deaths per year
Male life expectancy (0 years lag)							
*1* 37	serious^a^	serious^b^	not serious	not serious	none	⨁⨁◯◯ Low	−0.17 [−0.31, −0.02] years change in male life expectancy per year
Male life expectancy (5 years lag)							
*1* 37	serious^a^	serious^b^	not serious	not serious	none	⨁⨁◯◯ Low	−0.07 [−0.2, 0.06] years change in male life expectancy per year
Female life expectancy (0 years lag))							
*1* 37	serious^a^	serious^b^	not serious	not serious	none	⨁⨁◯◯ Low	−0.15 [−0.26, −0.04] years change in female life expectancy per year
Female life expectancy (5 years lag)							
*1* 37	serious^a^	serious^b^	not serious	not serious	none	⨁⨁◯◯ Low	−0.06 [−0.26, 0.01] years change in female life expectancy per year

^a^ Risk of bias as Assessed using ROBINS-I tool was judged to be moderate^34^ (“Crucial limitation for one criterion, or some limitations for multiple criteria, sufficient to lower confidence in the estimate of effect”).

^b^ We downgraded the quality of evidence on the grounds of inconsistency, as the majority but not all estimates showed the same direction of effect of austerity on mortality outcomes. Some effect estimates also had 95% confidence intervals which included no effect, but we did not downgrade for imprecision as well, so as to avoid double penalization.^52^

^c^ We employed a −3.2 unit shift in the cyclically adjusted primary balance (CAPB) for our analysis. This adjustment mirrors the alteration in the CAPB that the UK underwent in 2010, marking the initial year of deliberate policy shifts toward austerity in the country.

⨁◯◯◯ - Very low ⨁⨁◯◯ - Low ⨁⨁⨁◯ - Moderate ⨁⨁⨁⨁ - High

To put this into context, we used the CAPB measure of austerity to provide estimates of the effect of an increase in austerity as illustrated in Table 3. Based on the effects of a −3.2 unit change in CAPB, which mirrors the austerity transition experienced by the United Kingdom in 2010, the ASMR at a 0-year lag is estimated to see an increase of 115,385 additional deaths per year, (95% CI: 26,324 to 204,446). At a 5-year lag, there are an estimated 74,090 additional deaths per year (95% CI: 40,632 to 188,792). In terms of male life expectancy, there is an estimated reduction of 0.17 years at a 0-year lag (95% CI: −0.31 to −0.02). At a 5-year lag, there is an estimated reduction of 0.07 years (95% CI: −0.2 to 0.06). For female life expectancy at a 0-year lag, there is an estimated reduction of 0.15 years (95% CI: −0.26 to −0.04). This trend slightly moderates to a decline of 0.06 years (95% CI: −0.26 to 0.01) by a 5-year lag. These outcomes emphasize the potential negative effects of austerity, as captured by CAPB, on both ASMR and life expectancy over varied time lags. The absolute effect estimates of a unit increase in other austerity measures on mortality outcomes are illustrated in the appendix.

Given the low certainty of the evidence, further research is needed to better understand the impacts of different forms of austerity and the contextual interactions to strengthen our conclusions and provide more precise estimates of the health impacts of austerity.

## Discussion

This systematic review found consistent evidence of a relationship between austerity policies and harmful impacts on mortality outcomes in high-income countries, regardless of the measure of austerity used. Effects on lifespan variation were not uniform, varying with the specific austerity measure used, gender, and time lag. Data uncertainty arose from several limitations, such as the aggregation of diverse policy approaches into singular measures of austerity and the possible varying effects of austerity based on different economic contexts. The studies’ focus on overall austerity, rather than departmental-specific cuts, and their inability to distinguish between changes in tax and spending, as well as their distributive aspects, contributed to the uncertainty. The comparison of outcomes segregated by austerity measures was challenging due to issues of scale, as a unit change is not equivalent across different measures.

The effect sizes for austerity may be influenced by the inherent risk of residual confounding in observational studies, even when considering the most specific measures of overall austerity (the AAFI and CAPB) and accounting for most of the key confounding variables in the panel analysis approach. As a result, while there may be evidence for the presence of an effect, the exact magnitude of this effect remains uncertain.

The key strengths of this study include a rigorous and systematic search process, independent screening, and the use of reproducible methods to synthesize compatible effect estimates. Despite the heterogeneity of mortality outcomes, our methods allowed us to estimate the direction of the effect of austerity policies on mortality, even in the absence of meta-analysis. We followed recommended guidelines, such as the Cochrane handbook for systematic reviews of interventions and the ROBINS-I tool for assessing risk of bias. Finally, we used the GRADE approach to assess the overall certainty of our synthesized findings.

However, we only included English language articles, studies measuring overall fiscal austerity (as opposed to changes in funding for specific services that might have been introduced as part of an austerity program), and only studies with mortality outcomes. All studies apart from one included data from European countries only, limiting the generalizability beyond. Even the most specific measures of overall austerity that we identified (the AAFI and CAPB) may not adequately capture the policy intention behind austerity policies. This is for two reasons: first, during a period of rapid economic growth, a fiscal surplus in line with a Keynesian economic approach may not necessitate tax increases or expenditure cuts; however, the AAFI and CAPB measures could still classify this situation as austerity. One study did examine the specific impacts of austerity being implemented during periods of economic downturn, thereby removing the impacts of a Keynesian approach (of paying down government debt during periods of economic growth). This analysis found substantially larger and consistently harmful impacts of austerity on mortality and life expectancy.^50^ Second, fiscal deficits (i.e., non-austerity periods, or fiscal stimulus) may result from tax reductions for the richest groups, or increased spending on areas that may not enhance social welfare (e.g., military spending). As such, changes in the composition of tax and spending decisions may be at least as important as the fiscal balance (after accounting for automatic stabilizers). Again, the included studies in this review did not distinguish in their austerity measures between austerity implemented as changes in tax or changes in spending, nor in the distributive aspects of changes. Other analyses have considered the impacts of changes in austerity measured more specifically (e.g., reductions in public social spending and local government expenditure), finding consistently harmful impacts.^19,50^ Some approaches to austerity (such as in the United Kingdom) actually reduce the automatic stabilizers in the economy (e.g., by reducing the real value of social security and lowering tax rates), making adjustments for these mechanisms more difficult. For future studies, it may be worthwhile to consider alternative measures of austerity that incorporate a more nuanced approach, such as a combination of qualitative assessments of government policy changes and quantitative methods based on clusters of multiple variables. This would allow for a more comprehensive understanding of austerity and its impacts, while still maintaining a quantitative foundation.^53^

The limited number of studies included in this systematic review reflects exclusion of studies which did not examine the exact exposure of interest. The intention of this study was to examine the impact of overall fiscal austerity, as opposed to departmental-specific cuts to public spending. This focus was considered important and justified because, following the 2008 economic recession, governments exhibited varying responses in their policy decisions. Some initially pursued measures such as quantitative easing to maintain public spending in line with a Keynesian approach, while others, like the U.K. government from 2010 onwards, opted for an overall austerity agenda. Indeed, one of the included studies performed sensitivity analyses which provided effect sizes for the implementation of austerity during periods of economic downturn, finding even larger damaging effects on mortality and life expectancy.^50^ This might have led to an underestimate of the impact of austerity policies in the United Kingdom after 2010. These divergent policy choices highlight the significance of examining the impact of broad austerity measures on population health. As recession looms for many countries and consideration of austerity is becoming salient again, evidence regarding the impact of this broad policy approach may be valuable for governments. We therefore excluded less specific, but potentially still informative studies, which considered the impact of reduced public spending within specific sectors on population health. For example, several primary studies looked at the impact of specific policies (as opposed to overall fiscal austerity) on mortality outcomes, which tend to echo our findings suggestive of adverse impacts.^18,19,50,54,55^

Examples of studies that were screened but ultimately excluded include Alexiou and colleagues, who investigated the impact of government health expenditure on health outcomes using data from 15 G-20 countries between 2000 and 2018, finding a reduction in infant mortality rates with increased health expenditure.^56^ Alexiou and colleagues also examined the impact of local government spending in England on life expectancy outcomes, finding that cuts in funding for local government between 2013 and 2017 were associated with a decrease in life expectancy and an increase in premature mortality, particularly in more deprived areas, indicating that these cuts may have contributed to widening health inequalities.^19^ Another study by Darlington-Pollock and colleagues explored the impact of fiscal austerity policies on mortality rates in the United Kingdom following the 2008 financial recession.^57^ They found that the most deprived areas and those affected by fiscal austerity measures experienced the greatest excess deaths. Despite the potential relevance of these studies to the current review, they were excluded as they did not investigate the impact of overall fiscal austerity, instead focusing on area-specific cuts to public spending. Other studies were excluded because they examined the effects of austerity policies on non-mortality outcomes, such as poor mental health.^58^

One potential limitation of our use of ROBINS-I to assess studies using population-level data is the challenge of controlling for time-varying confounding. Given the inherent limitations of population-level studies in controlling for time-varying confounding, it is likely that even the best designed observational studies will be classified as having at least moderate risk of bias due to confounding. This is particularly relevant for studies investigating the effects of austerity policies on mortality outcomes, as the impact of these policies is likely to be influenced by a wide range of factors that vary over time. As a result, it can be difficult to distinguish between papers of the same level of bias in terms of their overall quality, leading to a lack of granularity in bias assessments.

Finally, although lifespan variation offers a proxy of socioeconomic inequalities in mortality, there were no explicit measures of the impact of austerity on mortality-related inequalities within countries. Future studies explicitly considering these inequalities in impacts would be of value. Despite these limitations, the panel analysis approach used by most of the studies to account for time invariant factors and the use of measures of austerity sensitive to automatic stabilizers accounted for most of the key confounding variables identified in our initial formulation. It would be difficult to further reduce the risks of confounding in future studies of this question. It has been suggested by some authors that time-varying factors such as influenza^59^ might be more important (and confound any relationship between austerity and mortality). However, subsequent data linkage work for England suggests that influenza deaths have not made a larger contribution over time,^3^ and the sustained stalling in the trends before and after the influenza epidemic in 2015 makes the influenza hypothesis highly unlikely.^60^ Indeed, if influenza were to be playing a role, it is most likely to be a mechanism linking austerity and mortality (e.g., through reductions in social care provision, reduced real incomes).

Although numerous studies have investigated the impact of economic recession on mortality outcomes, this is the first systematic review specifically examining the quantitative impact of overall austerity on mortality outcomes. Recession and austerity are distinct phenomena, each with potentially important and far-reaching, though not necessarily similar, impacts on population health. It is important, therefore, that they are considered distinctly, and that economic policies implemented in response to economic recessions are appraised for their health impacts. The small number of studies identified, explicitly examining the effects of overall austerity as opposed to discrete areas of public funding cuts on mortality outcomes, demonstrates the need for further research in this area.

Our review has important implications for policy, practice, and future research. We provide further evidence of the harmful effect of austerity policies on mortality. The available evidence suggests that austerity policies may be important in explaining the stalled trends in life expectancy across many high-income countries over the last decade. This is consistent with previous research examining the effect of cuts to specific public sector services on mortality outcomes.^17^ A review from Public Health England regarding stalled mortality trends from 2011 onwards suggested such trends were likely multifactorial, but failed to consider austerity as a possible cause.^3,11^ Further high-quality quantitative studies examining the relationship between austerity policies and mortality outcomes in a range of geopolitical contexts are needed. Future reviews could consider including a broader range of health outcomes and better measures of overall austerity, including the timing of austerity in relation to economic growth and recession; whether austerity was dominated by changes in taxes or spending; and the distributional nature of austerity, as well as specific austerity-associated policy changes, such as changes to social security or local government funding. This is particularly pertinent in the context of the global SARS-CoV-2 pandemic, which is forecasted to have impacts on the global economy matching or even exceeding those of the 2008 recession.^61^ As such, policymakers must remain cognizant of the evidence regarding the economic and public health validity of fiscal policy decisions and act to ensure that economic responses do not exacerbate health declines that have occurred throughout the pandemic.

## Supplemental Material

sj-docx-1-joh-10.1177_27551938241255041 - Supplemental material for Is Austerity Responsible for the Stalled Mortality Trends Across Many High-Income Countries? A Systematic Review Supplemental material, sj-docx-1-joh-10.1177_27551938241255041 for Is Austerity Responsible for the Stalled Mortality Trends Across Many High-Income Countries? A Systematic Review by Philip Broadbent, David Walsh, Srinivasa Vittal Katikireddi, Christine Gallagher, Ruth Dundas and Gerry McCartney in International Journal of Social Determinants of Health and Health Services
