# Supplementary material for: Is Austerity Responsible for the Stalled Mortality Trends Across Many High-Income Countries? A Systematic Review
Source: Int J Soc Determinants Health Health Serv. 2024 May 20;54(4):362–79. doi: 10.1177/27551938241255041 (PMC11437704; doi:10.1177/27551938241255041)
Supplement: sj-docx-1-joh-10.1177_27551938241255041 - Supplemental material for Is Austerity Responsible for the Stalled Mortality Trends Across Many High-Income Countries? A Systematic Review [file sj-docx-1-joh-10.1177_27551938241255041.docx]

Contents

[Appendix I – Search strategies 2](#_Toc139373677)

[Database(s): APA PsycInfo 2](#_Toc139373678)

[Database(s): ASSIA 3](#_Toc139373679)

[Database(s): **Cochrane** 3](#_Toc139373680)

[Database(s): **Cochrane** 4](#_Toc139373681)

[Database(s): **Cochrane** 5](#_Toc139373682)

[Database(s): OVID Medline 5](#_Toc139373683)

[Database(s): **Proquest PH** 6](#_Toc139373684)

[Database(s): **Scopus** 7](#_Toc139373685)

[Database(s): Sociological abstracts 7](#_Toc139373686)

[Database(s): Web of Science 7](#_Toc139373687)

[Appendix II – Search results 8](#_Toc139373688)

[Appendix III – Excluded studies 9](#_Toc139373689)

[Appendix IV - Data Extraction items 27](#_Toc139373690)

[Appendix V - ROBiNS-I Appraisals of included studies 28](#_Toc139373691)

[Appendix VI – PROSPERO protocol and revision history 35](#_Toc139373692)

## Appendix I – Search strategies

### Database(s): APA PsycInfo

Search Strategy:

| **#** | **Searches** | **Results** |
| --- | --- | --- |
| 1 | (public adj2 spend*).tw. | 309 |
| 2 | austerity.tw. | 895 |
| 3 | “fiscal austerity”.tw. | 38 |
| 4 | “fiscal stimulus”.tw. | 2 |
| 5 | (government adj2 expenditure).tw. | 93 |
| 6 | “spending cut*”.tw. | 39 |
| 7 | (public adj2 expenditure).tw. | 205 |
| 8 | “fiscal consolidation”.tw. | 5 |
| 9 | (government adj2 spend*).tw. | 234 |
| 10 | (state adj2 spend*).tw. | 106 |
| 11 | (state adj2 expenditure*).tw. | 98 |
| 12 | Public Expenditures/ | 0 |
| 13 | “Cyclically Adjusted Primary Balance”.tw. | 0 |
| 14 | “Alesina Ardagna Fiscal Index”.tw. | 0 |
| 15 | “Blanchard Fiscal Index”.tw. | 0 |
| 16 | “OECD Mean”.tw. | 1 |
| 17 | “life expectanc*”.tw. | 4957 |
| 18 | mortalit*.tw. | 42322 |
| 19 | “lifespan variation”.tw. | 5 |
| 20 | survival.tw. | 41548 |
| 21 | death*.tw. | 94796 |
| 22 | fatalit*.tw. | 3340 |
| 23 | dying.tw. | 12046 |
| 24 | “loss of life”.tw. | 469 |
| 25 | “years of life lost”.tw. | 201 |
| 26 | Life Expectancy/ | 3399 |
| 27 | exp Mortality/ | 40780 |
| 28 | Survival/ | 863 |
| 29 | exp Death/ | 0 |
| 30 | 1 or 2 or 3 or 4 or 5 or 6 or 7 or 8 or 9 or 10 or 12 or 13 or 14 or 15 or 16 | 1755 |
| 31 | 17 or 18 or 19 or 20 or 21 or 22 or 23 or 24 or 25 or 26 or 27 or 28 or 29 | 172928 |
| 32 | 30 and 31 | 125 |
| 33 | limit 32 to english language | 120 |

### Database(s): ASSIA

Search Strategy:

(noft(government NEAR/2 (spending* OR expenditure)) OR austerity OR “fiscal austerity” OR “fiscal stimulus” OR (“spending cut” OR “spending cuts”) OR “public expenditure” OR “fiscal consolidation” OR (“public spending”) OR “Cyclically Adjusted Primary Balance” OR “Alesina Ardagna Fiscal Index” OR “Blanchard Fiscal Index” OR “OECD mean”) AND noft((“life expectancies” OR “life expectancy”) OR mortalit* OR “lifespan variation” OR survival OR death* OR fatalit* OR dying OR “loss of life” OR “years of life lost” OR “Mortality Rates” OR longevity)

### Database(s): Cochrane

Search Strategy:

ID Search Hits

#1 (austerity) 22

#2 “fiscal austerity” 0

#3 “fiscal stimulus” 0

#4 “Spending cut*” 0

#5 “public expenditure” 7

#6 “fiscal consolidation” 0

#7 spend* 2728

#8 “public spend*” 0

#9 MeSH descriptor: [Public Expenditures] explode all trees 0

#10 “Cyclically Adjusted Primary Balance” 0

#11 “Alesina Ardagna Fiscal Index” 0

#12 “Blanchard Fiscal Index” 0

#13 “OECD Mean” 0

#14 #1 or #2 or #3 or #4 or #5 or #6 or #7 #9 or #10 or #11 or #12 or #13 29

#15 “life expectanc*” 1

#16 mortalit* 97607

#17 “lifespan variation” 0

#18 survival 109667

#19 death* 77621

#20 fatalit* 3468

#21 dying 1587

#22 “loss of life” 30

#23 “years of life lost” 54

#24 MeSH descriptor: [Life Expectancy] explode all trees 125

#25 MeSH descriptor: [Mortality] explode all trees 13304

#26 MeSH descriptor: [Survival] explode all trees 127

#27 MeSH descriptor: [Death] explode all trees 2196

#28 #15 or #16 or #17 or #18 or #19 or #20 or #21 or #22 or #23 or #24 or #25 or #26 or #27 212341

#29 #14 and #28 16

### Database(s): Cochrane

Search Strategy:

| **#** | **Searches** | **Results** |
| --- | --- | --- |
| 1 | (public adj2 spend*).tw. | 771 |
| 2 | austerity.tw. | 1115 |
| 3 | “fiscal austerity”.tw. | 52 |
| 4 | “fiscal stimulus”.tw. | 3 |
| 5 | (government adj2 expenditure).tw. | 403 |
| 6 | “spending cut*”.tw. | 90 |
| 7 | (public adj2 expenditure).tw. | 865 |
| 8 | “fiscal consolidation”.tw. | 8 |
| 9 | (government adj2 spend*).tw. | 454 |
| 10 | (state adj2 spend*).tw. | 144 |
| 11 | (state adj2 expenditure*).tw. | 231 |
| 12 | Public Expenditures/ | 165 |
| 13 | “Cyclically Adjusted Primary Balance”.tw. | 3 |
| 14 | “Alesina Ardagna Fiscal Index”.tw. | 1 |
| 15 | “Blanchard Fiscal Index”.tw. | 0 |
| 16 | “OECD Mean”.tw. | 2 |
| 17 | “life expectanc*”.tw. | 47897 |
| 18 | mortalit*.tw. | 1180960 |
| 19 | “lifespan variation”.tw. | 48 |
| 20 | survival.tw. | 1455607 |
| 21 | death*.tw. | 1217932 |
| 22 | fatalit*.tw. | 37172 |
| 23 | dying.tw. | 45832 |
| 24 | “loss of life”.tw. | 1488 |
| 25 | “years of life lost”.tw. | 2180 |
| 26 | Life Expectancy/ | 51088 |
| 27 | exp Mortality/ | 1131305 |
| 28 | Survival/ | 311530 |
| 29 | exp Death/ | 737085 |
| 30 | 1 or 2 or 3 or 4 or 5 or 6 or 7 or 8 or 9 or 10 or 12 or 13 or 14 or 15 or 16 | 3670 |
| 31 | 17 or 18 or 19 or 20 or 21 or 22 or 23 or 24 or 25 or 26 or 27 or 28 or 29 | 3771507 |
| 32 | 30 and 31 | 727 |
| 33 | limit 32 to english language | 695 |
| 34 | limit 33 to embase | 397 |

### Database(s): Cochrane

Search Strategy:

(austerity|“fiscal authority”|”fiscal stimulus”|”spending cut*”|”public expenditure”|”fiscal consolidation”|”public spend*”) AND (“life expectancy”|mortality|death|lifespan|dying)

First 50 results imported into Zotero via SciWheel

### Database(s): OVID Medline

Search Strategy:

| **#** | **Searches** | **Results** |
| --- | --- | --- |
| 1 | austerity.mp. | 964 |
| 2 | “fiscal austerity”.mp. | 54 |
| 3 | “fiscal stimulus”.mp. | 9 |
| 4 | “spending cut*”.mp. | 89 |
| 5 | “public expenditure”.mp. | 353 |
| 6 | “fiscal consolidation”.mp. | 9 |
| 7 | recession*.mp. | 13112 |
| 8 | Economic Recession/ | 2544 |
| 9 | Public Expenditures/ | 15 |
| 10 | “Cyclically Adjusted Primary Balance”.mp. | 3 |
| 11 | “Alesina Ardagna Fiscal Index”.mp. | 1 |
| 12 | “Blanchard Fiscal Index”.mp. | 0 |
| 13 | “OECD Mean”.mp. | 1 |
| 14 | 1 or 2 or 3 or 4 or 5 or 6 or 7 or 8 or 9 or 10 or 11 or 12 or 13 | 14223 |
| 15 | “life expectanc*”.mp. | 43219 |
| 16 | mortalit*.mp. | 1209496 |
| 17 | “lifespan variation”.mp. | 52 |
| 18 | survival.mp. | 1296039 |
| 19 | death*.mp. | 953964 |
| 20 | fatalit*.mp. | 30149 |
| 21 | dying.mp. | 36160 |
| 22 | “loss of life”.mp. | 1167 |
| 23 | “years of life lost”.mp. | 1806 |
| 24 | Life Expectancy/ | 17819 |
| 25 | exp Mortality/ | 393530 |
| 26 | Survival/ | 4780 |
| 27 | exp Death/ | 151023 |
| 28 | 15 or 16 or 17 or 18 or 19 or 20 or 21 or 22 or 23 or 24 or 25 or 26 or 27 | 2819206 |
| 29 | 14 and 28 | 1077 |

### Database(s): Proquest PH

Search Strategy:

(noft(government NEAR/2 (spending* OR expenditure)) OR austerity OR “fiscal austerity” OR “fiscal stimulus” OR (“spending cut” OR “spending cuts”) OR “public expenditure” OR “fiscal consolidation” OR (“public spending”) OR “Cyclically Adjusted Primary Balance” OR “Alesina Ardagna Fiscal Index” OR “Blanchard Fiscal Index” OR “OECD mean”) AND noft(“life expectanc*” OR mortalit* OR “lifespan variation” OR survival OR death* OR fatalit* OR dying OR “loss of life” OR “years of life lost” OR “Mortality Rates” OR longevity)

### Database(s): Scopus

Search Strategy:

TITLE-ABS-KEY (austerity OR “fiscal austerity” OR “fiscal stimulus” OR “spending cut*” OR “public expenditure” OR “fiscal consolidation” OR “public spend*” OR “Cyclically Adjusted Primary Balance” OR “Alesina Ardagna Fiscal Index” OR “Blanchard Fiscal Index” OR “OECD mean”) AND TITLE-ABS-KEY “life expectanc*” OR mortalit* OR “lifespan variation” OR survival OR death* OR fatalit* OR dying OR “loss of life” OR “years of life lost”) AND PUBYEAR > 2008 AND ( LIMIT-TO ( DOCTYPE , “ar” )) AND ( LIMIT-TO ( LANGUAGE , “English” ))

### Database(s): Sociological abstracts

Search Strategy:

(noft(government NEAR/2 (spending* OR expenditure)) OR austerity OR “fiscal austerity” OR “fiscal stimulus” OR (“spending cut” OR “spending cuts”) OR “public expenditure” OR “fiscal consolidation” OR (“public spending”) OR spend* OR “Cyclically Adjusted Primary Balance” OR “Alesina Ardagna Fiscal Index” OR “Blanchard Fiscal Index” OR “OECD mean”) AND noft(“life expectanc*” OR mortalit* OR “lifespan variation” OR survival OR death* OR fatalit* OR dying OR “loss of life” OR “years of life lost” OR “Mortality Rates” OR longevity)

### Database(s): Web of Science

Search Strategy:

TI=(“Public Expenditure” OR austerity OR “fiscal austerity” OR “fiscal stimulus” OR “spending cut*” OR “public expenditure” OR “fiscal consolidation” OR “public spend*” OR “Cyclically Adjusted Primary Balance” OR “Alesina Ardagna Fiscal Index” OR “Blanchard Fiscal Index” OR “OECD mean”) AND TI=(“Life expectancy” OR death OR “life expectanc*” OR mortalit* OR “lifespan variation” OR survival OR death* OR fatalit* OR dying OR “loss of life” OR “years of life lost”)

# Appendix II – Search results

| **Database** | **Results Jan 2021** | **Results Oct 2022** |
| --- | --- | --- |
| Medline | 396 | 121 |
| Embase | 322 | 117 |
| APA Psycinfo | 108 | 15 |
| Proquest Public Health | 1012 | 225 |
| Cochrane | 21 | 36 |
| Campbell | 0 | 0 |
| Web of Science | 43 | 7 |
| ASSIA | 211 | 44 |
| Sociological Abstracts | 1411 | 261 |
| Scopus | 388 | 64 |
| Google Scholar | 500 | 100 |

# Appendix III – Excluded studies

Wrong Exposure - (Does not include qualitative measure of austerity or proxy)

| **Author(s)** | **Year** | **Title** | **Journal** |
| --- | --- | --- | --- |
| Abbas, F., Awan, H. | 2018 | What determines health status of population in Pakistan? | Social Indicators Research |
| Aisa, R., Clemente, J., Pueyo, F. | 2014 | The influence of (public) health expenditure on longevity | International Journal of Public Health |
| Alex, C., Maresso, K. | 2014 | The impact of the financial crisis on the health system and health in Greece | Denmark Observatory on Health Systems and Policies |
| Alexander, M., Harding, L. | 2011 | Quantifying the impact of economic crises on infant mortality in advanced economies | Applied Economics |
| Alexiou, C., Trachanas, E. | 2021 | Politics, government health expenditure and infant mortality: Does political party orientation matter? | International Journal of Social Economics |
| Alvarez-Galvez, J., Salinas-Perez, J., Rodero-Cosano, M. | 2017 | Methodological barriers to studying the association between the economic crisis and suicide in Spain | BMC Public Health |
| Alvarez-Galvez, J., Suarez-Lledo, V., Salvador-Carulla, L., Almenara-Barrios, J. | 2021 | Structural determinants of suicide during the global financial crisis in Spain: Integrating explanations to understand a complex public health problem | PLoS One |
| Angelos, M., Maria, K. | 2013 | The impact of crisis on the health of citizens and in healthcare: The case of Greece | The Journal of Macro Trends in Health and Medicine |
| Arca, E., Principe, F. | 2020 | Death by austerity? The impact of cost containment on avoidable mortality in Italy | Health Economics |
| Azevedo, H., Constantino, S. | 2014 | The impact of the financial crisis on the health system and health in Portugal | European Observatory on Health Systems and Policies |
| Backhaus I., Hoven H., Di Tecco C., Iavicoli S., Conte A., Dragano N. | 2022 | Economic change and population health: Lessons learnt from an umbrella review on the Great Recession | BMJ Open |
| Blackman, T. | 2014 | Austerity and health | Dialogues in Human Geography |
| Borra, C., Pons-Pons, J. | 2020 | Austerity, healthcare provision and health outcomes in Spain | The European Journal of Health Economics |
| Branas, C., Kastakani, A. | 2015 | The impact of economic austerity and prosperity events on suicide in Greece: A 30-year interrupted time-series analysis | BMJ Open |
| Bruning, M., Thuilliez, J. | 2019 | Mortality and macroeconomic conditions: What can we learn from France? | Demography |
| Castro, M., Massudo, A., Almeida, G. | 2019 | Brazil’s unified health system: The first 30 years and prospects for the future | The Lancet |
| Chuang, Y., Chuang, K., You-Rong, S. | 2012 | Welfare state regimes, infant mortality and life expectancy: Integrating evidence from East Asia | Journal of Epidemiology and Community Health |
| Corcoran, P., Griffin, E., Arensman, E. | 2015 | Impact of the economic recession and subsequent austerity on suicide and self-harm in Ireland: An interrupted time series analysis | International Journal of Epidemiology |
| Cunningham, S. | 2009 | Causes of fluctuating mortality in Romania | European Journal of Population Health |
| Darlington-Pollock, F., Green, M., Simpson, L. |  | Why were there 231 707 more deaths than expected in England between 2010 and 2018? An ecological analysis of mortality records | Journal of Public Health |
| DeVogli, R. | 2013 | Financial crisis, austerity, and health in Europe | The Lancet |
| DeVogli, R. | 2011 | Neoliberal globalisation and health in a time of economic crisis | Social Theory & Health |
| Demakakos, P. | 2019 | Austerity, socioeconomic inequalities and stalling life expectancy in the UK: Two parallel stories or one? | Maturitas |
| Dorling, D., Rigby, J. | 2019 | Recession, austerity and life expectancy | Irish Medical Journal |
| Falagas, M., Vouloumanou, E., Mavros, M. | 2019 | Economic crises and mortality: A review of the literature | International Journal of Clinical Practice |
| Fountoulakis, K., Theodorakis, P. | 2014 | Austerity and health in Greece | The Lancet |
| Framklin, B., Hochlaf, D., Holley-Moore, G. | 2017 | Public Health in Europe during the austerity years | Research Report from ILC-UK |
| Frasquilho, D., Matos, M., Salonna, F. | 2015 | Mental health outcomes in times of economic recession: A systematic literature review | BMC Public Health |
| Gili, M., Roca, M., Basu, S. | 2013 | The mental health risks of economic crisis in Spain: Evidence from primary care centres 2006 and 2010 | The European Journal of Public Health |
| Goldstein, N., Palumbo, A., Bellamy, S. | 2020 | State and Local government expenditures and infant mortality in the United States | Pediatrics |
| Tapia Granados, J. | 2013 | A flawed diagnosis | Economics and Health |
| Holland, W. | 2012 | Austerity: A failed experiment on the people of Europe | Journal of the Royal College of Physicians |
| Holz, M., Mayerl, J. | 2021 | Early days of the pandemic—The association of economic and socio-political country characteristics with the development of the COVID-19 death toll | PLoS One |
| Ifanti, A., Argyriou, A., Kalofonou, F. | 2013 | Financial crisis and austerity measures in Greece: Their impact on health promotion policies and public health care | Health Policy |
| Maresso, A., Mladovsky, P., Thomson, S. | 2015 | Economic crisis, health systems and health in Europe | Observatory Studies Series |
| Jager, Philipp., Schmidt, T. | 2016 | The political economy of public investment when population is aging: A panel cointegration analysis | European Journal of Political Economy |
| Jones, R. | 2015 | Did austerity cause the rise in deaths seen in England and Wales in 2015 | British Journal of Health Care Management |
| Jones, R. | 2019 | Austerity in the UK and poor health: Were deaths directly affected? | British Journal of Health Care Management |
| Karanikolos, M., Mladovsky, P., Cylus, J. | 2013 | Financial crisis, austerity and health in Europe | The Lancet |
| Karim, W., Courtin, E., Muennig, P. | 2021 | Addressing the Social Determinants of Health in the Aftermath of COVID-19: Lessons From the 2008 Great Recession | American Journal of Public Health |
| Kentikelenis, A., Stubbs, T. | 2021 | Austerity redux: the post‐pandemic wave of budget cuts and the future of global public health | Global Policy |
| Kondilis, E., Giannakopoulos, S., Gavana, M. | 2013 | Economic crisis, restrictive policies and the populations health and health care: The Greek case | American Journal of Public Health |
| Kubrin, C., Bartos, B, McCleary, R. | 2022 | The debt crisis, austerity measures, and suicide in Greece | Social Science Quarterly |
| Langthorne, M. | 2019 | Austerity then and now | Health in Hard Times: Austerity and Health Inequalities |
| Lopez-Valcaracel, B., Beatriz, G., Barber, P. | 2017 | Economic crisis, austerity policies, health and fairness: lessons learned in Spain | Applied Economics and Health Policy |
| Mackenzie, T., Houle, J., Jiang, S. | 2019 | Middle-aged death and taxes in the USA: Association of state tax burden and expenditures in 2005 with survival from 2006 to 2015 | Plos One |
| Malta, D., Duncan, B., Barros, M. | 2018 | Fiscal austerity measures hamper noncommunicable disease control goals in Brazil | Ciencia & Saude Coletiva |
| Martin, S., Longo, F., Lomas, J., Claxton, K. | 2021 | Causal impact of social care, public health and healthcare expenditure on mortality in England: cross-sectional evidence for 2013/2014 | Bmj Open |
| Martins, T. | 2021 | Estimating the effects of fiscal consolidations: A synthetic control approach | Thesis, University of Lisbon |
| McCartney, G., Hearty, W., Arnot, J. | 2019 | Impact of political economy on population health: A systematic review of reviews | American Journal of Public Health |
| McKee, M., Karanikolos, M., Belcher, P. | 2012 | Austerity: a failed experiment on the people of Europe | Clinical Medicine |
| Morabia, A. | 2018 | Austerity policies and mortality in Spain after the financial crisis of 2008 | American Journal of Public Health |
| Neumayer, E. | 2004 | Recessions lower (some) mortality rates: evidence from Germany | Social Science and Medicine |
| Noy, S. | 2021 | For the children? A mixed methods analysis of world bank structural adjustment loans, health projects and infant mortality in Latin America | Globalisation and Health |
| Ortiz, I., Cummins, M. | 2013 | Austerity measures in developing countries: public expenditure trends and the risks to children and women | Feminist Economics |
| Paes-Sousa, R., Schramm, J., Mendes, L. | 2019 | Fiscal austerity and the health sector: the cost of adjustments | Ciencia & Saude Coletiva |
| Palma, M., Hernandez, I., Alvarez-Dardet, C. | 2009 | Economic factors related to the millennium development goals: a literature review | Revista Panamericana de Salud Publica |
| Park, D., Han, J., Torabi, M. | 2020 | Managing mental health: why we need to redress the balance between healthcare spending and social spending | BMC Public Health |
| Parmar, D., Stavropoulou, C., Ioannidis, J. | 2016 | Health outcomes during the 2008 financial crisis in Europe: systematic literature review | BMJ |
| Pierrakos, G., Balourdos, D., Soulis, S. | 2014 | Comparative analysis and evaluation of the effectiveness of demographic policies in EU countries (2009-2010) | World Health & Population |
| Popov, V. | 2012 | Russia: austerity and deficit reduction in historical and comparative perspective | Cambridge Journal of Economics |
| Puls H.T., Hall M., Anderst J.D., Gurley T., Perrin J., Chung P.J. | 2021 | State spending on public benefit programs and child maltreatment | Pediatrics |
| Rachiotis, G. | 2016 | The impact of economic crises on health: An overview | Disasters: Mental Health Context and Response |
| Rajmil, L., Siddiqi, A., Taylor-Robinson, D. | 2015 | Understanding the impact of the economic crisis on child health: the case of Spain | International Journal for Equity in Health |
| Regidor, E., Mateo, A., Barrio, G. | 2019 | Mortality in Spain in the context of the economic crisis and austerity policies | American Journal of Public Health |
| Reynolds, M. | 2018 | Health care public sector share and the US life expectancy lag: a country level longitudinal study | International Journal of Health Services: Planning, Administration, Evaluation |
| Reynolds, M . | 2018 | Social policy expenditures and life expectancy in high income countries | American Journal of Preventive Medicine |
| Rezaei, S., Oradi, K., Martin, B. | 2015 | Macro determinants of infant mortality in ECO countries: evidence from panel data analysis | International Journal of Paediatrics |
| Richardson, E., Taulbut, M., Robinson, M. | 2020 | The contribution of changes to tax and social security to stalled life expectancy trends in Scotland: a modelling study | Journal Of Epidemiology and Community Health |
| Roberts, A., Charlesworth, A. | 2014 | A decade of austerity in Wales | Nuffield Trust – Evidence for Better Healthcare |
| Roberts, A., Marshall, L. | 2010 | A decade of austerity | Nuffield Trust – Evidence for Better Healthcare |
| Ruckert, A., Labonte, R. | 2017 | Health inequities in the age of austerity: the need for social protection policies | Social Science and Medicine |
| Saez, M., Barcelo, M., Saurina, C. | 2019 | Evaluation of the biases in the studies that assess the effects of the great recession on health: a systematic review | International Journal of Environmental Research and Public Health |
| Schrecker, T., Bambra, C. | 2015 | Austerity: how politics has pulled away our safety net | How Politics Makes Us Sick (Book) |
| Sharma, A. | 2020 | Does economic freedom improve health outcomes in sub-Saharan Africa | International Journal of Social Economics |
| Sherpa, D. | 2020 | Estimating impact of austerity policies in COVID-19 fatality rates: examining the dynamics of economic policy and case fatality rates of Covid-19 in OECD countries | SSRN Journal |
| Shiroyama T., Fukuyama K., Okada M. | 2021 | Effects of financial expenditure of prefectures/municipalities on regional suicide mortality in Japan | International Journal of Environmental Research and Public Health |
| Simou, E., Koutsogeorgou, E. | 2014 | Effects of the economic crisis on health and healthcare in Greece in the literature from 2009 to 2013: a systematic review | Health Policy |
| Sommer, J. | 2020 | Accountable government spending: A cross-national analysis of child mortality in developing nations | International Journal of Health Services: Planning, Administration, Evaluation |
| Stevens, A., Miller, D., Page, M. | 2015 | The best of times, the worst of times: understanding pro-cyclical mortality | American Economic Journal |
| Stuckler, D., Basu, S. | 2013 | The body economic: why austerity kills | Book |
| Stuckler, D., Dasu, S. | 2013 | The body economic: eight experiments in economic recovery, from Iceland to Greece | Book |
| Stuckler, D., Reeves, A., Loopstra, R. | 2018 | Austerity and health: the impact of crisis in the UK and the rest of Europe | Book |
| Stuckler, D., Basu, S., Suhrcke, M. | 2009 | The public health effect of economic crises and alternative policy responses in Europe: an empirical analysis | The Lancet |
| Stuckler, D., McKee, M. | 2012 | There is an alternative: public health professionals must not remain silent at a time of financial crisis | Oxford University Press |
| Suhrcke, M., Stuckler, D. | 2010 | Will the recession be bad for our health? | European Centre for Health Assets and Architecture |
| Sun, T., Tao, R., Su, C., Umar, M. | 2021 | How Do Economic Fluctuations Affect the Mortality of Infectious Diseases? | Frontiers in Public Health |
| Tapia-Granados, J., Rodriguez, J. | 2015 | Health, economic crisis and austerity: A comparison of Greece, Finland and Iceland | Health Policy |
| Taylor-Robinson, D., Gosling, R., Harrison, D. | 2013t | Austerity measures hit the sickest hardest | BMJ |
| Taylor-Robinson, D., Bradshaw, J., Whitehead, M. | 2014 | Child mortality in the UK | The Lancet |
| Therborn, Goran. | 2009 | The killing fields of inequality | Soundings |
| Triantafyllou, K., Angeletopoulou, C. | 2011 | Increased suicidality amid economic crisis in Greece | Lancet Correspondence |
| Tuttle, J. | 2018 | Specifying the effect of social welfare expenditures on homicide and suicide: a cross-national, longitudinal examination of the stream analogy of lethal violence | Justice Quarterly |
| Tyrovolas, S., Kassebaum, N., Stergachis, A. | 2018 | The burden of disease in Greece, health loss, risk factors, and health financing, 2000-16: an analysis of the Global burden of disease study 2016 | The Lancet Public Health |
| Van Gool, K., Pearson, M. | 2014 | health, austerity and economic crisis: assessing the short-term impact in OECD countries | OECD library |
| Vizard, P., Obolenskaya, P. | 2015 | The coalition’s record on health: Policy, spending and outcomes 2010-2015 | Social Policy in a Cold Climate Working Paper |
| Vogt, T., Kluge, F. | 2015 | Can public spending reduce mortality disparities? Findings from East Germany after reunification | The Journal of the Economics of Ageing |
| Vrachnis, N., Vlachadis, N., Iliodromiti, Z. | 2014 | Austerity and health in Greece | The Lancet |
| Vrachnis, N., Vlachadis, N., Iliodromiti, Z. | 2014 | Austerity and health | The Lancet |
| Woolf, S. | 2011 | Public health implications of government spending reductions | Journal of the American Medical Association |
| Yakubu, Y., Nor, N., Abidin, E. | 2018 | A systematic review of micro correlates of maternal mortality | Reviews on Environmental Health |
| Zavras, D., Tsiantou, V., Pavi, E. | 2013 | Impact of economic crisis and other demographic and socio-economic factors on self-rated health in Greece | The European Journal of Public Health |
| Zilidis, C., Stuckler, D., McKee, M. | 2020 | Use of amenable mortality indicators to evaluate the impact of financial crisis on health system performance in Greece | European Journal of Public Health |
| International Centre for Longevity UK | 2021 | Tippinig the scales: Exploring austerity and public health in the UK | Working paper |
| Public Health Wales Observatory | 2020 | Life expectancy & mortality in Wales | Working paper |
| Institute of Health Equity | 2020 | Health equity in England: The Marmot review 10 years on | Working paper |

**Wrong Study Design**

| **Author(s)** | **Year** | **Title** | **Journal** |
| --- | --- | --- | --- |
| Adam, S., Papatheodorou, C. | 2016 | Dismantling the feeble social protection system of Greece: consequences of the crisis and austerity measures | Challenges to European Welfare Systems |
| Beckfield, J., Krieger, N. | 2009 | Epi + demos + cracy: Linking political systems and priorities to the magnitude of health inequities – evidence, gaps, and a research agenda | Epidemiological Reviews |
| Cabrera de Leon, A., Rodriguez, I., Gannar F | 2018 | Austerity Policies and mortality in Spain after the financial crisis of 2008 | American Journal of Public Health |
| Collins, C. | 2019 | Austerity and Mortality in Spain: The perils of overcorrecting an analytic mistake | American Journal of Public Health |
| Collucci, C. | 2018 | Brazil’s child and maternal mortality have increased against background of public spending cuts | BMJ (Clinical Research Education) |
| DeVogli, R. | 2011 | Neoliberal globalisation and health in a time of economic crisis | Social Theory & Health |
| Kim, D. | 2011 | Do USA state-level social spending and income inequality predict individual mortality? A fixed effects, instrumental variable analysis. | Journal of Epidemiology and Community Health |
| Kim, I., Muntaner, C., Vahid, S. | 2012 | Welfare states, flexible employment and health: a critical review | Health Policy |
| McCartney, G., Hearty, W., Arnot, J. | 2019 | Impact of political economy on population health: A systematic review of reviews | American Journal of Public Health |
| Modrek, S., Stuckler, D., McKee, M. | 2013 | A review of health consequences of recessions internationally and a synthesis of the US response during the great recession | Public Health Reviews |
| Morabia, A. | 2018 | Austerity policies and mortality in Spain after the financial crisis of 2008 | American Journal of Public Health |
| Parmar, D., Stavropoulou, C., Ioannidis, J. | 2016 | Health outcomes during the 2008 financial crisis in Europe: systematic literature review | BMJ |
| Popov, V. | 2012 | Russia: austerity and deficit reduction in historical and comparative perspective | Cambridge Journal of Economics |
| Stuckler, D., Dasu, S. | 2013 | The body economic: eight experiments in economic recovery, from Iceland to Greece | Book |
| Stuckler, D., Basu, S., McKee, M. | 2010 | Protection and public health: population evidence from the EU 1980-2003 | Journal of Epidemiology and Community Health |
| Tapia-Granados, J., Rodriguez, J. | 2015 | Health, economic crisis and austerity: A comparison of Greece, Finland and Iceland | Health Policy |
| Woolf, S. | 2011 | Public health implications of government spending reductions | Journal of the American Medical Association |

**Wrong Population: Not national-level population/not high-income country**

| **Author(s)** | **Year** | **Title** | **Journal** |
| --- | --- | --- | --- |
| Goldstein, N., Palumbo, A., Bellamy, S. | 2020 | State and Local government expenditures and infant mortality in the United States | Pediatrics |
| Malta, D., Duncan, B., Barros, M. | 2018 | Fiscal austerity measures hamper noncommunicable disease control goals in Brazil | Ciencia & Saude Coletiva |
| Sharma, A. | 2020 | Does economic freedom improve health outcomes in sub-Saharan Africa | International Journal of Social Economics |

**Wrong Outcome: Not mortality-derived measure**

| **Author(s)** | **Year** | **Title** | **Journal** |
| --- | --- | --- | --- |
| Liu, L. | 2013 | The austerity trap: economic and social consequences of fiscal consolidation in Europe | Academia.edu |
| Martins, T. | 2021 | Estimating the effects of fiscal consolidations: A synthetic control approach | Thesis, University of Lisbon |
| Ortiz, I., Cummins, M. | 2013 | Austerity measures in developing countries: public expenditure trends and the risks to children and women | Feminist Economics |
| Rajmil, L., Siddiqi, A., Taylor-Robinson, D. | 2015 | Understanding the impact of the economic crisis on child health: the case of Spain | International Journal for Equity in Health |
| Sherpa, D. | 2020 | Estimating impact of austerity policies in COVID-19 fatality rates: examining the dynamics of economic policy and case fatality rates of Covid-19 in OECD countries | SSRN Journal |

# Appendix IV - Data Extraction items

Data extracted for each paper included the following

| **Item** | **Possible values** |
| --- | --- |
| Reference | #1–#5 |
| Authors | Author surnames (Free-text) |
| Title | Title of study (Free-text) |
| Publication Year | Year of Publication (Free-text) |
| Study Design | E.g., Panel Analysis, Cross-sectional analysis, Longitudinal Study |
| Population(s) | Population type (country level? Number of countries?) |
| Time Period | Years included in study |
| Measurement of Exposure Variable | Metric used to measure austerity (e.g., CAPB, AAFI, TGE as a percentage of GDP) |
| Scale and duration of Austerity measure | Continuous/Categorical |
| Comparator | What comparator was used (e.g., between populations? Same population over different time periods) |
| Measurement of Outcome variable | Metric used to measure mortality (e.g., life expectancy, all-cause standardized mortality rate, excess mortality) |
| Effect of change in measure of austerity on measure of mortality outcome | Quantitative measure of impact of austerity on mortality |
| To what degree are conclusions supported by analysis and are the statistical tests used appropriate? | Comment on analysis techniques and statistical measures used? (Free-text) |

# Appendix V - ROBiNS-I Appraisals of included studies

| **Signalling question** | **McCartney**  **(2022)** | **Predkiewciz**  **(2022)** | **Rajmil (2019)** | **Toffoluti (2019)** | **Rajmil (2018)** | **Antonokakis (2015)** | **Green (2017)** |
| --- | --- | --- | --- | --- | --- | --- | --- |
| **1.1 Is there potential for confounding of the effect of intervention (exposure) in this study?** | PY | PY | Y | PY | Y | Y | Y |
| **1.2. Was the analysis based on splitting follow up time according to intervention (intervention (exposure)) received?** | PN | PN | N | N | N | N | N |
| **1.3. Were intervention (exposure) discontinuations or switches likely to be related to factors that are prognostic for the outcome?** | NA | NA | NA | NA | NA | NA | NA |
| **1.4. Did the authors use an appropriate analysis method that adjusted for all the critically important confounding domains?** | Y | Y | PN | PN | N | N | N |
| **1.5. Were confounding areas that were adjusted for measured validly and reliably by the variables available in this study?** | PY | PN | Y | Y | Y | Y | NA |
| **1.6. Did the authors avoid adjusting for post-intervention (exposure) variables?** | Y | Y | N | N | N | N | NA |
| **1.7. Did the authors use an appropriate analysis method that adjusted for all the critically important confounding areas and for time-varying confounding?** | NA | NA | N | N | N | N | N |
| **1.8. Were confounding areas that were adjusted for measured validly and reliably by the variables available in this study?** | NA | NA | NA | NA | NA | NA | NA |
| **Risk Of Bias Judgement** | Moderate | Moderate | Moderate | Moderate | Serious | Critical | Critical |
|  |  |  |  |  |  |  |  |
| **2.1. Was selection of participants (populations) into the study (or into the analysis) based on variables measured after the start of the intervention (exposure)?** | N | N | PN | N | PN | N | N |
| **2.2. Were the post-intervention (exposure) variables that influenced selection associated with intervention (exposure)?** | NA | NA | NA | NA | NA | NA | NA |
| **2.3 Were the post-intervention (exposure) variables that influenced eligibility selection influenced by the outcome or a cause of the outcome?** | NA | NA | NA | NA | NA | NA | NA |
| **2.4. Do start of follow-up and start of intervention (exposure) coincide for most participants?** | Y | Y | Y | Y | Y | Y | Y |
| **2.5. Were adjustment techniques used that are likely to correct for the presence of selection biases?** | NA | NA | NA | NA | NA | NA | NA |
| **Risk of Bias Judgement** | Low | Low | Low | Low | Low | Low | Low |
|  |  |  |  |  |  |  |  |
| **3.1 Is intervention (exposure) status well defined?** | Y | PN | PY | Y | PY | Y | N |
| **3.2 Was information used to define intervention (exposure)) status recorded at the start of the intervention (exposure)?** | N | N | Y | Y | Y | Y | PY |
| **3.3 Could classification of intervention (exposure) status have been affected by knowledge of the outcome or risk of the outcome?** | N | N | N | N | N | NA | N |
| **Risk of Bias Judgement** | Low | Moderate | Low | Low | Low | Low | Serious |
|  |  |  |  |  |  |  |  |
| **4.1. Were there deviations from the intended intervention (exposure) beyond what would be expected in usual practice?** | N | N | N | N | N | N | N |
| **4.2. Were these deviations from intended intervention (exposure) unbalanced between groups and likely to have affected the outcome?** | NA | NA | NA | NA | NA | NA | NA |
| **4.3. Were important co-interventions (or exposures) balanced across intervention groups?** | Y | Y | NA | NA | NA | NA | NA |
| **4.4. Was the intervention (exposure) implemented successfully for most participants?** | Y | Y | NA | NA | NA | Y | Y |
| **4.5. Did study participants adhere to the assigned intervention (exposure) regimen?** | Y | Y | NA | NA | NA | Y | Y |
| **4.6. an appropriate analysis used to estimate the effect of starting and adhering to the intervention (exposure)?** | NA | NA | NA | NA | NA | NA | NA |
| **Risk of Bias Judgement** | Low | Low | Low | Low | Low | Low | Low |
|  |  |  |  |  |  |  |  |
| **5.1 Were data available for all, or nearly all, participants?** | Y | N | Y | Y | Y | Y | Y |
| **5.2 Were participants excluded due to missing data on intervention (exposure) status?** | N | N | N | N | N | N | N |
| **5.3 Were participants excluded due to missing data on other variables needed for the analysis?** | N | N | N | N | N | N | N |
| **5.4. Are the proportion of participants and reasons for missing data similar across intervention (exposure)s?** | NA | NA | NA | NA | NA | NA | NA |
| **5.5. Is there evidence that results were robust to the presence of missing data?** | NA | NA | NA | NA | NA | NA | NA |
| **Risk of Bias Judgement** | Low | Moderate | Low | Low | Low | Low | Low |
|  |  |  |  |  |  |  |  |
| **6.1 Could the outcome measure have been influenced by knowledge of the intervention (exposure) received?** | N | N | N | N | N | N | N |
| **6.2 Were outcome assessors aware of the intervention (exposure) received by study participants?** | N | N | PN | N | PN | N | N |
| **6.3 Were the methods of outcome assessment comparable across intervention (exposure) groups?** | Y | Y | Y | Y | Y | Y | Y |
| **6.4 Were any systematic errors in measurement of the outcome related to intervention (exposure) received?** | N | N | N | N | Y | PN | PN |
| **Risk of Bias Judgement** | Low | Low | Low | Low | Serious | Low | Low |
|  |  |  |  |  |  |  |  |
| **7.1. Is the reported effect estimate likely to be selected, on the basis of the results, from multiple outcome measurements within the outcome domain?** | N | N | N | N | N | Y | N |
| **7.2. Is the reported effect estimate likely to be selected, on the basis of the results, from multiple analyses of the intervention (exposure)-outcome relationship?** | N | N | N | N | N | N | N |
| **7.3. Is the reported effect estimate likely to be selected, on the basis of the results, from different subgroups?** | N | N | N | N | N | N | N |
| **Risk of Bias Judgement** | Low | Low | Low | Low | Low | Critical | Low |
|  |  |  |  |  |  |  |  |
| **Overall RoB Judgement** | Moderate | Moderate | Moderate | Moderate | Serious | Critical | Critical |
|  |  |  |  |  |  |  |  |

# Appendix VI – Expanded GRADE assessment of certainty of direction of effect of austerity on mortality outcomes table

| **Certainty assessment** | | | | | | | | | **Summary of findings** |  |  |  |  |  |  |  |  |  |
| --- | --- | --- | --- | --- | --- | --- | --- | --- | --- | --- | --- | --- | --- | --- | --- | --- | --- | --- |
| ***Number of studies***  **Countries (observations)** | **Risk of bias** | **Inconsistency** | **Indirectness** | | **Imprecision** | **Publication bias** | | **Overall certainty of evidence** | Absolute effect estimates on mortality outcomes of a -3.2 unit in CAPB^c^ when applied to the ONS UK Population estimate (mid year 2021) of 67,026,292 [95%CI] |  | |  |  |  |  |  |  |  |
|  |  |  |  |  |  |  |  |  |  |  | |  |  |  |  |  |  |  |
| **Age standardized mortality rate (0 years lag)** | | | | | | | | | |  |  |  |  |  |  |  |  |  |
| *1*  37 (1) | serious^a^ | serious^b^ | not serious | | not serious | none | | ⨁⨁◯◯ Low | 115,385 [26,324 – 204,446] additional deaths per year |  |  |  |  |  |  |  |  |  |
| **Age standardized mortality rate (5 Years lag)** | | | | | | | | | | |  |  |  |  |  |  |  |  |
| *1*  37 (1) | serious^a^ | serious^b^ | | not serious | not serious | | none | ⨁⨁◯◯ Low | 74,090[-40632 – 188792] additional deaths per year | |  |  |  |  |  |  |  |  |
| **Male life expectancy (0 years lag)** | | | | | | | | | |  |  |  |  |  |  |  |  |  |
| *1*  37 (1) | serious^a^ | serious^b^ | not serious | | not serious | none | | ⨁⨁◯◯ Low | -0.17 [-0.31 - -0.02] years |  |  |  |  |  |  |  |  |  |
| **Male life expectancy (5 years lag)** | | | | | | | | | |  |  |  |  |  |  |  |  |  |
| *1*  37 (1) | serious^a^ | serious^b^ | not serious | | not serious | none | | ⨁⨁◯◯ Low | -0.07 [-0.2 – 0.06] years |  |  |  |  |  |  |  |  |  |
| **Female life expectancy (0 years lag))** | | | | | | | | | |  |  |  |  |  |  |  |  |  |
| *1*  37 (1) | serious^a^ | serious^b^ | not serious | | not serious | none | | ⨁⨁◯◯ Low | -0.15[-0.26 - -0.04] years |  |  |  |  |  |  |  |  |  |
| **Female life expectancy (5 years lag)** | | | | | | | | | |  |  |  |  |  |  |  |  |  |
| *1*  37 (1) | serious^a^ | serious^b^ | not serious | | not serious | none | | ⨁⨁◯◯ Low | -0.06[-0.26 – 0.01] years |  |  |  |  |  |  |  |  |  |
| ***Number of studies***  **Countries (observations)** | **Risk of bias** | **Inconsistency** | **Indirectness** | | **Imprecision** | **Publication bias** | | **Overall certainty of evidence** | Absolute effect estimates on mortality outcomes of a -1.28 unit in AAFI^d^ when applied to the ONS UK Population estimate (mid year 2021) of 67,026,292 [95%CI] |  |  |  |  |  |  |  |  |  |
|  |  |  |  |  |  |  |  |  |  |  |  |  |  |  |  |  |  |  |
| **Age standardized mortality rate (0 years lag)** | | | | | | | | | |  |  |  |  |  |  |  |  |  |
| *1*  37 (1) | serious^a^ | serious^b^ | not serious | | not serious | none | | ⨁⨁◯◯ Low | 46,154 [10,529 – 81,778] additional deaths per year |  |  |  |  |  |  |  |  |  |
| **Age standardized mortality rate (5 years lag)** | | | | | | | | | | |  |  |  |  |  |  |  |  |
| *1*  37 (1) | serious^a^ | serious^b^ | | not serious | not serious | | none | ⨁⨁◯◯ Low | 27,088 [-22,852 – 77, 036] additional deaths per year | |  |  |  |  |  |  |  |  |
| **Male life expectancy (0 years lag)** | | | | | | | | | |  |  |  |  |  |  |  |  |  |
| *1*  37 (1) | serious^a^ | serious^b^ | not serious | | not serious | none | | ⨁⨁◯◯ Low | 0.00 [-0.08 – 0.07] years |  |  |  |  |  |  |  |  |  |
| **Male life expectancy (5 years lag)** | | | | | | | | | |  |  |  |  |  |  |  |  |  |
| *1*  37 (1) | serious^a^ | serious^b^ | not serious | | not serious | none | | ⨁⨁◯◯ Low | -0.03 [-0.09 - -0.02] years |  |  |  |  |  |  |  |  |  |
| **Female life expectancy (0 years lag))** | | | | | | | | | |  |  |  |  |  |  |  |  |  |
| *1*  37 (1) | serious^a^ | serious^b^ | not serious | | not serious | none | | ⨁⨁◯◯ Low | 0.00 [-0.01 – 0.01] years |  |  |  |  |  |  |  |  |  |
| **Female life expectancy (5 years lag)** | | | | | | | | | |  |  |  |  |  |  |  |  |  |
| *1*  37 (1) | serious^a^ | serious^b^ | not serious | | not serious | none | | ⨁⨁◯◯ Low | -0.03 [ -0.11 – 0.03] years |  |  |  |  |  |  |  |  |  |
| ***Number of studies***  **Countries (observations)** | **Risk of bias** | **Inconsistency** | **Indirectness** | | **Imprecision** | **Publication bias** | | **Overall certainty of evidence** | Absolute effect estimates on mortality outcomes of a -2.76 unit in Government spending as a percentage of GDP^e^ when applied to the ONS UK Population estimate (mid year 2021) of 67,026,292 [95%CI] |  |  |  |  |  |  |  |  |  |
|  | | | | | | | | | |  | | |  |  |  |  |  |  |
| **Age standardized mortality rate (0 years lag)** | | | | | | | | | |  |  |  |  |  |  |  |  |  |
| *1*  37 (1) | serious^a^ | serious^b^ | not serious | | not serious | none | | ⨁⨁◯◯ Low | 181,985 [175,052 – 190,651] additional deaths per year |  |  |  |  |  |  |  |  |  |
| **Age standardized mortality rate (5 years lag)** | | | | | | | | | | |  |  |  |  |  |  |  |  |
| *1*  37 (1) | serious^a^ | serious^b^ | | not serious | not serious | | none | ⨁⨁◯◯ Low | 5199 [-27,731 – 15,598] additional deaths per year | |  |  |  |  |  |  |  |  |
| **Male Life expectancy (0 years lag)** | | | | | | | | | |  |  |  |  |  |  |  |  |  |
| *1*  37 (1) | serious^a^ | serious^b^ | not serious | | not serious | none | | ⨁⨁◯◯ Low | -0.29 [-0.30 - -0.27] years |  |  |  |  |  |  |  |  |  |
| **Male life expectancy (5 years lag)** | | | | | | | | | |  |  |  |  |  |  |  |  |  |
| *1*  37 (1) | serious^a^ | serious^b^ | not serious | | not serious | none | | ⨁⨁◯◯ Low | 0.00 [-0.2 – 0.2] years |  |  |  |  |  |  |  |  |  |
| **Female life expectancy (0 years lag))** | | | | | | | | | |  |  |  |  |  |  |  |  |  |
| *1*  37 (1) | serious^a^ | serious^b^ | not serious | | not serious | none | | ⨁⨁◯◯ Low | -0.21 [-0.22 - -0.20] years |  |  |  |  |  |  |  |  |  |
| **Female life expectancy (5 years lag)** | | | | | | | | | |  |  |  |  |  |  |  |  |  |
| *1*  37 (1) | serious^a^ | serious^b^ | not serious | | not serious | none | | ⨁⨁◯◯ Low | 0.00 [-0.02 – 0.04] years |  |  |  |  |  |  |  |  |  |
| a. Risk of bias as assessed using RoBINS-I tool was judged to be “Moderate” (2) (“Crucial limitation for one criterion, or some limitations for multiple criteria, sufficient to lower confidence in the estimate of effect”).  b. We downgraded the quality of evidence on the grounds of inconsistency, as the majority but not all estimates showed the same direction of effect of austerity on mortality outcomes. Some effect estimates also had 95% confidence intervals which included no effect, but we did not downgrade for imprecision as well, so as to avoid “double penalization.”  c. We employed a -3.2 unit shift in the Cyclically Adjusted Primary Balance (CAPB) for our analysis. This adjustment mirrors the alteration in the CAPB that the UK underwent in 2010, marking the initial year of deliberate policy shifts toward austerity in the country.  d. We employed a -1.28 unit shift in the Alesia-Ardagna Fiscal Index (AAFI) for our analysis. This adjustment mirrors the alteration in the AAFI that the UK underwent in 2010, marking the initial year of deliberate policy shifts toward austerity in the country.  e. We employed a -2.76 shift in government spending as a percentage of GDP for our analysis. This adjustment mirrors the alteration in the Government spending as a percentage of GDP that the UK underwent in 2010, marking the initial year of deliberate policy shifts toward austerity in the country.  ⨁◯◯◯ - Very low ⨁⨁◯◯ - Low ⨁⨁⨁◯ - Moderate ⨁⨁⨁⨁ - High | | | | | | | | | |  |  |  |  |  |  |  |  |  |

# Appendix VII – PROSPERO protocol and revision history

**Revision history**

This protocol was first submitted to PROSPERO on 16th December 2020. Revisions were made on 18th February 2021 to clarify the inclusion and exclusion criteria after discussion following the initial scoping searches.

**Deviations from protocol**

Meta-analysis not possible within confines of results obtained.

Calculation of absolute effect estimates were not specific in the PROSPERO protocol but were included in the final manuscript.

**Review question**

To what extent do policies of fiscal austerity cause changes in population life expectancy and all-cause mortality?

**Searches**

Structured searches will be carried out in the following databases: MEDLINE (OVID), EmBase, PsycINFO, ProQuest Public Health Database, The Cochrane and Campbell Libraries Web of Science, ASSIA and PPH Databases, Social Services Abstracts and Sociological abstracts.

An initial search strategy will be designed for MEDLINE and adapted to suit individual databases. In addition to databases searches, snowballing searches (i.e. citations tracking; authors tracking; references list consultation) also will be conducted over the articles selected through the database searches.

In addition, we will manually search relevant sources of grey literature including: WHOInt, The Institute for Fiscal Studies (IFS), The Joseph Rowntree Foundation, The Food Foundation, OpenGrey Europe (www.opengrey.eu), Public Health England, Public Health Scotland, Public Health Wales, Centre for Disease Control, United Nations, Organisation for Economic Cooperation and Development. Documents relating to the UK government (using gov.uk/publications).

Finally we will search the first 50 web pages of Google Scholar using Boolean searches for keywords.

The systematic search will be conceptualized as below and as a combination of:

Austerity terms (any of these): “austerity,” “fiscal stimulus,” “government/state/public spending,” “government/state/public expenditure,” etc.

AND life expectancy and mortality terms (any of these):

“life expectancy,” “mortality,” “lifespan variation,” “survival,” “death,” “years of life lost”

**Condition or domain being studied**

The effect of fiscal austerity measures on life expectancy, all-cause mortality, survival and lifespan Variation, either for entire populations, population strata (age groups and sex groups), or inequalities in these measures.

**Population**

Any national level population, of any age group, at any time period post-financial crisis (from 2008 onwards) of affected high-income countries. We will define high-income countries according to the World bank definition as countries whose “economies are those with a GNI per capita of $12,536 or more.”

**Exposure(s)**

There is increasing interest, from both a research and a policy point of view, in the links between “Austerity” measures in high-income countries and the effects on Life Expectancy, mortality and lifespan variation. The definition of Austerity is ambiguous. Most definitions of Austerity share a commonality of “fiscal consolidation,” but Wren-Lewis argues that fiscal consolidation and austerity are not necessarily synonymous (Wren-Lewis S. 2016, A general theory of austerity). Instead, the term austerity should be reserved for when fiscal consolidation measures are applied during a downturn or recession, which are likely to further deflate demand. We will therefore take policies aimed at fiscal consolidation applied in the context of an economic recession as our definition of Austerity.

The most specific qualitative measures of austerity include Cyclically Adjusted Primary Balance (CAPB) and the Alesina-Ardagna Fiscal Index (also called “Blanchard Fiscal Index”) (6). However, based on initial scoping of the literature, very few studies employ these measures and the review would therefore be very limited in scope. As such we will expand our search to include proxies of austerity, such as reductions in total government expenditure (while excluding area-specific measures such as change in healthcare spending).

**Comparator/control**

We will use only studies including some form of comparison either:

- in the same population before and after implementation of austerity
- between countries employing differing degrees of austerity

**Types of study to be included**

Randomized and non-randomized quantitative studies. To include: Longitudinal studies; cross-sectional studies, natural experiment studies.

**Time frame**

We will consider all time periods.

**Countries of interest**

Research on World Bank defined high income countries only will be included.

**Main outcome(s)**

The aim of this research is to focus, as an initial step towards a broader understanding of the links between economic policy and inequality, the speculated association between Austerity Policies and Life Expectancy. Outcome measures for this study must include measures derived from mortality measures including:

- life expectancy
- all-cause or cause-specific mortality
- survival
- lifespan variation

Other measures such as morbidity, self-assessed health, well-being, illness, and ill health will not be included. Measures of effect Any.

**Additional outcome(s)**

Inequalities in the outcomes specified above.

**Data extraction**

Selection: studies relevant to the research question will be selected independently by two reviewers with disputes resolved by discussion. This will involve initial title and abstract screening followed by full-text screening against the inclusion criteria. For relevant reviews, the individual studies within the review will be screened against the inclusion criteria. The software program Covidence will be used to manage the process and to produce a PRISMA flowchart.

Extraction: narrative and quantitative data as available will be extracted in summarized form, as available. Data will be extracted into an Excel spreadsheet by one reviewer and checked by another. Disputes will be resolved by discussion.

The effect size from individual studies will be extracted alongside data on the context (country, population group, time period, austerity measure, etc.), the outcome measure(s) used, and the range of confounding factors that were adjusted for. Any information on the nature of the austerity (or counter-cyclical spending increase) will also be extracted (i.e., whether this consisted mainly of changes to spending or taxation and the distributional implications of any changes).

**Risk of bias (quality) assessment**

We intend to use ROBINS-I critical appraisal approach assessing the following domains:

***Pre-intervention***

- *Domain 1: confounding* – key confounders considered for the present study are discussed below
- *Domain 2: selection of participants into the study –* This involved consideration of the following: were countries selected according to appropriate selection criteria? Was selection of countries into the study based on characteristics apparent after the star of the intervention? Did follow-up coincide with the start of the exposure for included populations?

***At intervention***

- *Domain 3: classification of exposure* – This involved consideration of the following: Were exposure groups clearly defined? Could classification of the exposure status have been affected by knowledge of the outcome or risk of the outcome?

***Post-intervention***

- *Domain 4: deviation from intended exposure –* This involved consideration of the following: were the deviations in the exposure that were unanticipated? Were these deviations unbalanced between groups and likely to have affected outcome?
- *Domain 5: missing data* – This involved consideration of the following: were outcome data available for all included countries. This involved consideration of the following: Were countries excluded due to missing data on exposure status or other variables needed for the analysis?
- *Domain 6: measurement of outcomes –* This involved consideration of the following: could the outcome measure have been influenced by knowledge of the exposure received? Were there any systematic errors in measurement of the outcome related to the exposure received?
- *Domain 7: selection of reported result –* This involved consideration of the following: Is the reported effect estimate likely to be selected, on the basis of the results, from multiple outcome measurements within the outcome domain?

**Data synthesis**

If there are sufficient data to facilitate a meta-analysis this will be undertaken alongside checks for publication bias and study heterogeneity. If a meta-analysis is not possible for all the included studies, the data will also be synthesized narratively giving greater weight to the data from higher quality studies following the approach detailed in the SWiM guideline.

**Analysis of subgroups or subsets**

If there are sufficient data, we intend to perform secondary subgroup analysis stratifying information for each of the outcome domains both by type of austerity measure (e.g., increased taxation compared to reduction in welfare benefits) and, where possible, the level of austerity imposed. We will also seek to show any differences in effects by the mean age, sex distribution and prevalent poverty levels through stratified analyses, as these are our theorized effect modifiers. 30.

**Type and method of review**

Type of review: Systematic review

**Dissemination plans**

Academic publication. Dissemination through the Scottish Mortality Special Interest Group.

**Keywords**

Systematic review; austerity; health; mortality; high-income countries

# Appendix VII – GRADE certainty of evidence and absolute effect estimate table

| **Certainty assessment** | | | | | | | | | **Summary of findings** |  |  |  |  |  |  |  |  |  |
| --- | --- | --- | --- | --- | --- | --- | --- | --- | --- | --- | --- | --- | --- | --- | --- | --- | --- | --- |
| ***Number of studies***  **Countries (observations)** | **Risk of bias** | **Inconsistency** | **Indirectness** | | **Imprecision** | **Publication bias** | | **Overall certainty of evidence** | Absolute effect estimates on mortality outcomes of a -3.2 unit in CAPB^c^ when applied to the ONS UK Population estimate (mid-year 2021) of 67,026,292 [95% CI] |  | |  |  |  |  |  |  |  |
|  |  |  |  |  |  |  |  |  |  |  | |  |  |  |  |  |  |  |
| **Age standardized mortality rate (0 years lag)** | | | | | | | | | |  |  |  |  |  |  |  |  |  |
| *1*  37 (1) | serious^a^ | serious^b^ | not serious | | not serious | none | | ⨁⨁◯◯ Low | 115,385 [26,324 – 204,446] additional deaths per year |  |  |  |  |  |  |  |  |  |
| **Age standardized mortality rate (5 years lag)** | | | | | | | | | | |  |  |  |  |  |  |  |  |
| *1*  37 (1) | serious^a^ | serious^b^ | | not serious | not serious | | none | ⨁⨁◯◯ Low | 74,090 [-40632 – 188792] additional deaths per year | |  |  |  |  |  |  |  |  |
| **Male life expectancy (0 years lag)** | | | | | | | | | |  |  |  |  |  |  |  |  |  |
| *1*  37 (1) | serious^a^ | serious^b^ | not serious | | not serious | none | | ⨁⨁◯◯ Low | -0.17 [-0.31 - -0.02] years |  |  |  |  |  |  |  |  |  |
| **Male life expectancy (5 years lag)** | | | | | | | | | |  |  |  |  |  |  |  |  |  |
| *1*  37 (1) | serious^a^ | serious^b^ | not serious | | not serious | none | | ⨁⨁◯◯ Low | -0.07 [-0.2 – 0.06] years |  |  |  |  |  |  |  |  |  |
| **Female life expectancy (0 years lag))** | | | | | | | | | |  |  |  |  |  |  |  |  |  |
| *1*  37 (1) | serious^a^ | serious^b^ | not serious | | not serious | none | | ⨁⨁◯◯ Low | -0.15 [-0.26 - -0.04] years |  |  |  |  |  |  |  |  |  |
| **Female life expectancy (5 years lag)** | | | | | | | | | |  |  |  |  |  |  |  |  |  |
| *1*  37 (1) | serious^a^ | serious^b^ | not serious | | not serious | none | | ⨁⨁◯◯ Low | -0.06 [-0.26 – 0.01] years |  |  |  |  |  |  |  |  |  |
| ***Number of studies***  **Countries (observations)** | **Risk of bias** | **Inconsistency** | **Indirectness** | | **Imprecision** | **Publication bias** | | **Overall certainty of evidence** | Absolute effect estimates on mortality outcomes of a -1.28 unit in AAFI^d^ when applied to the ONS UK Population estimate (mid-year 2021) of 67,026,292 [95% CI] |  |  |  |  |  |  |  |  |  |
|  |  |  |  |  |  |  |  |  |  |  |  |  |  |  |  |  |  |  |
| **Age standardized mortality rate (0 years lag)** | | | | | | | | | |  |  |  |  |  |  |  |  |  |
| *1*  37 (1) | serious^a^ | serious^b^ | not serious | | not serious | none | | ⨁⨁◯◯ Low | 46,154 [10,529 – 81,778] additional deaths per year |  |  |  |  |  |  |  |  |  |
| **Age standardized mortality rate (5 years lag)** | | | | | | | | | | |  |  |  |  |  |  |  |  |
| *1*  37 (1) | serious^a^ | serious^b^ | | not serious | not serious | | none | ⨁⨁◯◯ Low | 27,088 [-22,852 – 77, 036] additional deaths per year | |  |  |  |  |  |  |  |  |
| **Male life expectancy (0 years lag)** | | | | | | | | | |  |  |  |  |  |  |  |  |  |
| *1*  37 (1) | serious^a^ | serious^b^ | not serious | | not serious | none | | ⨁⨁◯◯ Low | 0.00 [-0.08 – 0.07] years |  |  |  |  |  |  |  |  |  |
| **Male life expectancy (5 years lag)** | | | | | | | | | |  |  |  |  |  |  |  |  |  |
| *1*  37 (1) | serious^a^ | serious^b^ | not serious | | not serious | none | | ⨁⨁◯◯ Low | -0.03 [-0.09 - -0.02] years |  |  |  |  |  |  |  |  |  |
| **Female life expectancy (0 years lag))** | | | | | | | | | |  |  |  |  |  |  |  |  |  |
| *1*  37 (1) | serious^a^ | serious^b^ | not serious | | not serious | none | | ⨁⨁◯◯ Low | 0.00 [-0.01 – 0.01] years |  |  |  |  |  |  |  |  |  |
| **Female life expectancy (5 years lag)** | | | | | | | | | |  |  |  |  |  |  |  |  |  |
| *1*  37 (1) | serious^a^ | serious^b^ | not serious | | not serious | none | | ⨁⨁◯◯ Low | -0.03 [ -0.11 – 0.03] years |  |  |  |  |  |  |  |  |  |
| ***Number of studies***  **Countries (observations)** | **Risk of bias** | **Inconsistency** | **Indirectness** | | **Imprecision** | **Publication bias** | | **Overall certainty of evidence** | Absolute effect estimates on mortality outcomes of a -2.76 unit in Government spending as a percentage of GDP^e^ when applied to the ONS UK Population estimate (mid-year 2021) of 67,026,292 [95% CI] |  |  |  |  |  |  |  |  |  |
|  | | | | | | | | | |  | | |  |  |  |  |  |  |
| **Age standardized mortality rate (0 years lag)** | | | | | | | | | |  |  |  |  |  |  |  |  |  |
| *1*  37 (1) | serious^a^ | serious^b^ | not serious | | not serious | none | | ⨁⨁◯◯ Low | 181,985 [175,052 – 190,651] additional deaths per year |  |  |  |  |  |  |  |  |  |
| **Age standardized mortality rate (5 years lag)** | | | | | | | | | | |  |  |  |  |  |  |  |  |
| *1*  37 (1) | serious^a^ | serious^b^ | | not serious | not serious | | none | ⨁⨁◯◯ Low | 5199 [-27,731 – 15,598] additional deaths per year | |  |  |  |  |  |  |  |  |
| **Male life expectancy (0 years lag)** | | | | | | | | | |  |  |  |  |  |  |  |  |  |
| *1*  37 (1) | serious^a^ | serious^b^ | not serious | | not serious | none | | ⨁⨁◯◯ Low | -0.29 [-0.30 - -0.27] years |  |  |  |  |  |  |  |  |  |
| **Male life expectancy (5 years lag)** | | | | | | | | | |  |  |  |  |  |  |  |  |  |
| *1*  37 (1) | serious^a^ | serious^b^ | not serious | | not serious | none | | ⨁⨁◯◯ Low | 0.00 [-0.2 – 0.2] years |  |  |  |  |  |  |  |  |  |
| **Female life expectancy (0 years lag))** | | | | | | | | | |  |  |  |  |  |  |  |  |  |
| *1*  37 (1) | serious^a^ | serious^b^ | not serious | | not serious | none | | ⨁⨁◯◯ Low | -0.21 [-0.22 - -0.20] years |  |  |  |  |  |  |  |  |  |
| **Female life expectancy (5 years lag)** | | | | | | | | | |  |  |  |  |  |  |  |  |  |
| *1*  37 (1) | serious^a^ | serious^b^ | not serious | | not serious | none | | ⨁⨁◯◯ Low | 0.00 [-0.02 – 0.04] years |  |  |  |  |  |  |  |  |  |
| a. Risk of bias as Assessed using RoBINS-I tool was judged to be “Moderate” (2) (“Crucial limitation for one criterion, or some limitations for multiple criteria, sufficient to lower confidence in the estimate of effect”).  b. We downgraded the quality of evidence on the grounds of inconsistency, as the majority but not all estimates showed the same direction of effect of austerity on mortality outcomes. Some effect estimates also had 95% confidence intervals which included no effect, but we did not downgrade for imprecision as well, so as to avoid “double penalization.”  c. We employed a -3.2 unit shift in the Cyclically Adjusted Primary Balance (CAPB) for our analysis. This adjustment mirrors the alteration in the CAPB that the UK underwent in 2010, marking the initial year of deliberate policy shifts toward austerity in the country.  d. We employed a -1.28 unit shift in the Alesia-Ardagna Fiscal Index (AAFI) for our analysis. This adjustment mirrors the alteration in the AAFI that the UK underwent in 2010, marking the initial year of deliberate policy shifts toward austerity in the country.  e. We employed a -2.76 shift in government spending as a percentage of GDP for our analysis. This adjustment mirrors the alteration in the Government spending as a percentage of GDP that the UK underwent in 2010, marking the initial year of deliberate policy shifts toward austerity in the country.  ⨁◯◯◯ - Very low ⨁⨁◯◯ - Low ⨁⨁⨁◯ - Moderate ⨁⨁⨁⨁ - High | | | | | | | | | |  |  |  |  |  |  |  |  |  |
